# Using artificial intelligence to predict mortality in AKI patients: a systematic review/meta-analysis

**DOI:** 10.1093/ckj/sfae150

**Published:** 2024-05-17

**Authors:** Rupesh Raina, Raghav Shah, Paul Nemer, Jared Fehlmen, Lena Nemer, Ali Murra, Abhishek Tibrewal, Sidharth Kumar Sethi, Javier A Neyra, Jay Koyner

**Affiliations:** Akron Nephrology Associates/Cleveland Clinic Akron General Medical Center, Akron, OH, USA; Department of Nephrology, Akron Children's Hospital, Akron, OH, USA; Akron Nephrology Associates/Cleveland Clinic Akron General Medical Center, Akron, OH, USA; Northeast Ohio Medical University, Rootstown, OH, USA; Baylor College of Medicine, Houston, TX, USA; Northeast Ohio Medical University, Rootstown, OH, USA; University of Missouri-Kansas City School of Medicine, Kansas City, MO, USA; Northeast Ohio Medical University, Rootstown, OH, USA; Akron Nephrology Associates/Cleveland Clinic Akron General Medical Center, Akron, OH, USA; Pediatric Nephrology, Medanta, The Medicity, Gurgaon, Haryana, India; Department of Medicine, Division of Nephrology, University of Alabama at Birmingham, Birmingham, AL, USA; Section of Nephrology, Department of Medicine, University of Chicago, Chicago, IL, USA

**Keywords:** acute kidney injury, artificial intelligence, machine learning, mortality, prediction

## Abstract

**Background:**

Acute kidney injury (AKI) is associated with increased morbidity/mortality. With artificial intelligence (AI), more dynamic models for mortality prediction in AKI patients have been developed using machine learning (ML) algorithms. The performance of various ML models was reviewed in terms of their ability to predict in-hospital mortality for AKI patients.

**Methods:**

A literature search was conducted through PubMed, Embase and Web of Science databases. Included studies contained variables regarding the efficacy of the AI model [the AUC, accuracy, sensitivity, specificity, negative predictive value and positive predictive value]. Only original studies that consisted of cross-sectional studies, prospective and retrospective studies were included, while reviews and self-reported outcomes were excluded. There was no restriction on time and geographic location.

**Results:**

Eight studies with 37 032 AKI patients were included, with a mean age of 65.1 years. The in-hospital mortality was observed to be 19.8%. The pooled [95% confidence interval (CI)] AUC was observed to be highest for the broad learning system (BLS) model [0.852 (0.820–0.883)] and elastic net final (ENF) model [0.852 (0.813–0.891)], and lowest for proposed clinical model (PCM) [0.765 (0.716–0.814)]. The pooled (95% CI) AUC of BLS and ENF did not differ significantly from other models except PCM [Delong's test *P* = 0.013]. PCM exhibited the highest negative predictive value, which supports this model's use as a possible rule-out tool.

**Conclusion:**

Our results show that BLS and ENF models are equally effective as other ML models in predicting in-hospital mortality, with variability across all models. Additional studies are needed.

KEY LEARNING POINTS **What was known:**

- Acute kidney injury (AKI) in-hospital mortality rates have increased tremendously over the past decade.
- Many machine learning and artificial intelligence (AI) tools have been developed to predict mortality in patients with AKI.

**This study adds:**

- Using eight varying AI models used to predict mortality in patients with AKI, there was no clear discriminatory results between the models.

**Potential impact:**

- The results presented in this manuscript outline that clinicians should use AI models to predict AKI mortality with caution and be wary of potential biases and limitations, but also that they should use the tool that is most generalizable to their patient.

## INTRODUCTION

Acute kidney injury (AKI) is a condition characterized by a significant decline in kidney function that results in the dysfunction of the kidneys’ ability to properly filter waste products and regulate fluid/electrolyte homeostasis [1]. Beyond the acute phase of AKI, progression to chronic kidney disease, increased risk of cardiovascular complications, recurrent episodic AKI and long-term mortality are common in survivors of AKI [2, 3]. AKI affects approximately 15% of hospitalized patients and 30%–60% of patients in the intensive care unit (ICU), with high morbidity and mortality risk in both settings. Continuous renal replacement therapy (CRRT) is commonly used to provide renal support in ICU patients with severe AKI, yet the mortality rate of AKI patients receiving CRRT remains high at 30%–70% [4, 5]. Improvement in patient outcomes with AKI has proven to be complex due to the condition's wide array of etiologies and complex pathophysiology, which often leads to a delay in diagnosis [2].

Earlier identification of high-risk AKI patients can lead to better-targeted interventions that have the potential to reduce morbidity and mortality, contribute to more efficient resource allocation, decrease length of stay and reduce total hospital expenses for patients [6]. To improve the prediction of mortality in AKI patients, prior studies have evaluated prognostic models that employ both conventional statistical modeling techniques and advanced artificial intelligence (AI) modeling techniques [6].

Machine learning (ML) is a field of artificial intelligence (AI) that computes future predictions from previous input. These algorithms identify patterns, relationships and inputs from given data and then apply these externally to novel data [3]. Linear ML models such as logistic regression or linear regression aim to understand the relationships between variables and predict outcomes [8]. These linear ML models may be affected by factors of multicollinearity and non-linear, more complex relationships [6–10].

Advanced non-linear modeling techniques of ML such as random forest (RF), extreme gradient boosting (XGBoost), broad learning system (BLS), support vector machine (SVM), artificial neural network (ANN), multi-layer perceptron (MLP) and other AI models have emerged as promising prediction models due to their ability to factor in more complex variables. It is anticipated that these AI models will be able to enhance prognostic accuracy and overcome shortcomings of previous models [6]. By efficiently utilizing large datasets such as the Medical Information Mart for Intensive Care III and patient electronic health records, these models can discriminately predict new, complex clinical scenarios, thus reducing mortality and poor patient outcomes in AKI patients [4, 6]. Furthermore, even subjective factors such as sentiment analysis and nursing notes have shown to be highly useful in these advanced models [4].

The aim of this study was to compare the performance of different ML models and other AI models in predicting in-hospital mortality of patients with established AKI. Statistical analyses and performance metrics were compared to analyze the potential differences in predictors and predictive output of such models as well as the presence of heterogeneity and publication bias.

## MATERIALS AND METHODS

### Literature search

A literature search was conducted using databases including PubMed and Embase, by two independent reviewers. The search strategy was conducted using broader terminology specifically targeting AI and ML utilization in AKI, due to vastly limited results with using “mortality” or synonymous terms as a primary search query. Thus, primary search queries included “Artificial Intelligence,” “Machine Learning,” “Deep Learning” and “Acute Kidney Injury.” The search was not restricted to any age group. The search was restricted to English, and the selected articles were exported to a citation managing software “Rayyan.” All literature results were reviewed by two independent reviewers (R.S. and P.N.). Any disagreements regarding data extraction were resolved by a third reviewer (R.R.). A detailed search strategy can be found in the Supplementary data file.

### Selection criteria

The studies selected were appropriate for inclusion when statistical variables regarding the efficacy of the AI model were reported [the AUC, accuracy, sensitivity, specificity, negative predictive value (NPV) and positive predictive value (PPV)]. Additionally, only original studies that consisted of cross-sectional studies, prospective and retrospective studies were included in the systematic review. Furthermore, there was no restriction on time (given that most studies included were in the past decade) and geographic location. Publications classified as review articles, systematic reviews and self-reported outcomes were excluded from this meta-analysis. A detailed PICOS (population, intervention, comparator, outcome, study design) table can be found in the Supplementary data file.

### Data extraction

The data extraction of included articles was performed using a standardized data collection tool, which included first author, title, year of publication, sample size, gender distribution, mean age, geography and study design. ML/AI model specific characteristics consisted of AUC, accuracy, sensitivity, specificity, NPV and PPV. A detailed data extraction table can be found in the Supplementary data file.

### Results of literature search

From a total of 623 articles gathered after an initial search from multiple databases, only 8 articles matched our inclusion criteria and were utilized for meta-analysis. Qualitative analysis of the included studies was done using the Newcastle-Ottawa Scale (NOS) tool. The qualitative analysis can be found in the supplemental data file.

### Statistical analysis

The outcomes included model performance metrics (AUC, sensitivity/recall, specificity, PPV/precision, NPV and accuracy) of different ML/AI models in assessing in-hospital mortality among AKI patients. These outcomes and their 95% confidence intervals (95% CIs) were extracted for each of the included study. The degree of between-study heterogeneity was assessed using the I^2^ test, where I^2^ ≥ 50% indicated high heterogeneity. The overall (pooled) estimate was calculated using a random effects model for high heterogeneity and fixed effects model for low heterogeneity. Analysis of the performance of individual models was conducted. However, to simplify the complexity of comparing each set of results from each study's implementation of a model with another, pooling the resulting values presents a more efficient manner of comparing overall model performance results, which has also been conducted.

A forest plot was used to visualize these outcomes in each study and the combined estimated outcomes with their 95% CI. Publication bias was assessed with an Egger's test. The DeLong's test was used to compare the receiver operating characteristic curves of the two models. A *P*-value ≤.05 was set as the level of significance. All statistical analyses were performed with R software version 3.1.0.

## Results

### Included studies

A total of eight studies including patients with AKI admitted in an ICU/hospital were included. Of these eight studies, two included AKI patients undergoing CRRT, one included COVID-19 patients with AKI and one included patient with cardiac surgery–associated AKI. The overall sample size across these studies was 37 032 (ranging from 270 to 19 044 across various studies). The mean/median age of the patients across these studies was 65.1 years (range 62.1–77.7 years). The proportion of patients with in-hospital mortality was 19.8% (7348/37 032) among the included studies. Of the eight studies, two (*n* = 20 615) included only derivation/training cohorts, while the other six (*n* = 16 417) included both derivation/training (*n* = 12 200) and validation/testing (*n* = 4217) cohorts. Additional information about the presence or absence of derivation/validation cohorts can be found in the Supplementary data file.

### ML/AI models across different studies

A total of 14 different ML/AI models were reported in these studies. These included: logistic regression (four studies) [3, 6, 8, 11], RF (seven studies) [3–7, 10, 11], XGBoost (four studies) [3, 5, 10, 11], SVM (four studies) [3, 5, 7, 11], ANN/MLP (three studies) [3, 5, 7], BLS models (one study) [4], elastic net final (ENF) model fitted (one study) [10], PCM (which combined variables commonly selected with multiple ML methods plus clinical rationale of utility/feasibility assessment by the investigators) (one study) [11], κ-nearest neighbor (KNN) (one study) [5], multivariable adaptive regression splines (MARS) (one study) [5], customized Simplified Acute Physiology Score II (SAPS II) (one study) [7], mortality scoring system for acute kidney injury with CRRT (MOSAIC model) (one study) [5], Sequential Organ Failure Assessment (SOFA) (one study) [5] and Acute Physiologic Assessment and Chronic Health Evaluation II (APACHE II) (one study) [5].

### Area under the curve

**Figure 1:**
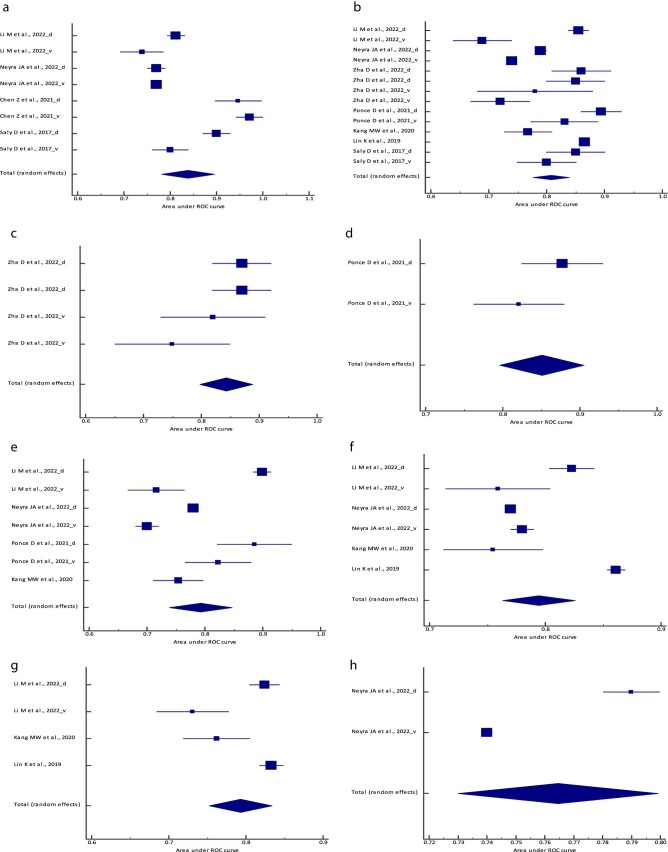
Forest plot of the meta-analysis of the (**a**) logistic regression model, (**b**) the RF model, (**c**) the BLS model, (**d**) the ENF model, (**e**) the XGBoost model, (**f**) the SVM model, (**g**) the ANN/MLP model and (**h**) the PCM AUC across different studies. The lower diamond in each graph represents the pooled estimate AUC.

All eight studies reported the data on AUC for 14 different ML/AI models. A meta-analysis was conducted for eight models with data from one or more cohort as shown in Table 1. Across these eight models, the pooled (95% CI) AUC was observed to be highest for BLS models [0.852 (0.820–0.833)] and elastic net final (ENF) model [0.852 (0.813–0.891)], and lowest for proposed clinic model (PCM) [0.765 (0.716–0.814)]. This difference was statistically significant (Delong's test *P *= .013). However, the AUC of BLS model was not significantly different from other models. The pooled (95% CI) AUC of logistic regression was observed to be higher than that of XGBoost, RF, SVM and ANN/MLP, but not significantly different from these models. There was no evidence of publication bias for most of the models based on Egger's test (*P* > .05) except ANN/MLP, ENF model fitted, and PCM. Tables 1–6 also provide detail on heterogeneity analysis for each model. Tables 7a–7h and Fig. 1 provide meta-analysis of AUC for individual ML/AI models across different studies.

**Table 1:** Meta-analysis of AUC for different ML/AI models in predicting in-hospital mortality among AKI patients.

Model	No. of studies; no. of cohorts	No. of mortality/sample size	Pooled AUC (95% CI)	I^2^ (95% CI); *P*-values	Egger's test (*P*-value)
BLS models^a^	1 study; 2 D and 2 V cohort	278/540	0.852 (0.820–0.883)	44.88% (0.00%–81.61%); *P *= .1421	.0526
ENF model fitted^a^	1 study; 1 D and 1 V cohort	544/870	0.852 (0.813–0.891)	48.05% (8.04%–75.25%); *P *= .1653	<.0001
Logistic regression	4 studies; 4 D and 4 V cohort	3056/15 277	0.837 (0.800–0.873)	97.8% (96.89%–98.44%); *P *< .0001	.0502
XGBoost	4 studies; 4 D and 3 V cohort	4356/15 229	0.793 (0.739–0.846)	98.04% (97.19%–98.63%); *P *< .0001	.6971
RF	7 studies; 8 D and 6 V cohort	7444/37 054	0.807 (0.763–0.852)	99.62% (99.56%–99.67%); *P *< .0001	.3367
SVM	4 studies; 4 D and 2 V cohort	6402/33 403	0.793 (0.753–0.833)	99.02% (98.66%–99.29%); *P *< .0001	.3719
ANN/MLP	3 studies; 3 D and 1 V cohort	4571/23 816	0.793 (0.752–0.833)	87.22% (69.38%–94.66%); *P *< .0001	.0085
PCM^b^	1 study; 1 D and 1 V cohort	1831/9587	0.765 (0.716–0.814)	98.96% (97.92%–99.48%); *P *< .0001	<.0001
Customized SAPS II	1 study; 1 D	2590/19 044	0.795 (0.781–0.809)	No meta-analysis done	
MARS	1 study; 1 D	1019/1571	0.756 (0.713–0.799)	No meta-analysis done	
KNN	1 study; 1 D	1019/1571	0.721 (0.675–0.767)	No meta-analysis done	
MOSAIC	1 study; 1 D	1019/1571	0.690 (0.641–0.740)	No meta-analysis done	
SOFA	1 study; 1 D	1019/1571	0.664 (0.636–0.691)	No meta-analysis done	
APACHE II	1 study; 1 D	1019/1571	0.593 (0.563–0.622)	No meta-analysis done	

a Fixed effect models, for other random effect models.

b This clinical model was restricted to 14–15 variables that were top features of different ML models and were deemed by the investigators as pragmatic given its routine use/access to in clinical practice.

No meta-analysis done as there was only one cohort.

D: derivation; V: validation.

**Table 2:** Meta-analysis of sensitivity of different ML/AI models in predicting in-hospital mortality among AKI patients.

Model	No. of studies; no. of cohorts	No. of mortality/sample size	Pooled sensitivity (95% CI)	I^2^ (95% CI); *P*-values	Egger's test (*P*-value)
RF	3 studies; 4 D and 4 V cohort	3071/13 328	0.73 (0.694–0.767)	86.77% (76.05%–92.69%); *P *< .0001	.6889
BLS models	1 study; 2 D and 2 V cohort	278/540	0.711 (0.516–0.906)	95.25% (90.77%–97.55%); *P *< .0001	.0509
SVM	2 studies; 2 D and 2 V cohort	2793/12 788	0.707 (0.634–0.781)	98.05% (96.77%–98.82%); *P *< .0001	.3671
PCM^a^	1 study; 1 D and 1 V cohort	1831/9587	0.706 (0.677–0.736)	86.11% (44.55%–96.52%); *P *= .0073	.0001
XGBoost	2 studies; 2 D and 2 V cohort	2793/12 788	0.698 (0.643–0.752)	95.82% (92.09%–97.79%); *P *< .0001	.7130
Logistic regression	2 studies; 2 D and 2 V cohort	2793/12 788	0.691 (0.629–0.752)	96.39% (93.35%–98.04%); *P *< .001	.7847
ANN/MLP	1 study; 1 D and 1 V cohort	962/3201	0.652 (0.591–0.713)	59.76% (0.00%–90.57%); *P *= .1149	.0001

Fixed effect models, for other random effect models.

a This clinical model was restricted to 14–15 variables that were top features of different ML models and were deemed by the investigators as pragmatic given its routine use/access to in clinical practice.

D: derivation; V: validation.

**Table 3:** Meta-analysis of specificity of different ML/AI models in assessing in-hospital mortality among AKI patients.

Model	No. of studies; no. of cohorts	No. of mortality/sample size	Pooled specificity (95% CI)	I^2^ (95% CI); *P*-values	Egger's test (*P*-value)
ANN/MLP	1 study; 1 D and 1 V cohort	962/3201	0.791 (0.727–0.855)	87.03% (49.07%–96.70%); *P *= .0055	<.0001
SVM	2 studies; 2 D and 2 V cohort	2793/12 788	0.736 (0.683–0.788)	96.81% (94.25%–98.23%); *P *< .0001	.9617
BLS models	1 study; 2 D and 2 V cohort	278/540	0.728 (0.478–0.978)	97.34% (95.36%–98.47%); *P *< .0001	.0675
XGBoost	2 studies; 2 D and 2 V cohort	2793/12 788	0.727 (0.656–0.799)	98.46% (97.54%–99.03%); *P *< .0001	.7396
Logistic regression	2 studies; 2 D and 2 V cohort	2793/12 788	0.724 (0.654–0.795)	97.32% (95.32%–98.46%); *P *< .0001	.9904
RF	3 studies; 4 D and 4 V cohort	3071/13 328	0.709 (0.656–0.762)	98.65% (98.19%–99.00%); *P *< .0001	.9491
PCM^a^	1 study; 1 D and 1 V cohort	1831/9587	0.675 (0.607–0.744)	99.47% (99.07%–99.70%); *P *< .0001	<.0001

Fixed effect models, for other random effect models.

a This clinical model was restricted to 14–15 variables that were top features of different ML models and were deemed by the investigators as pragmatic given its routine use/access to in clinical practice.

D: derivation; V: validation.

**Table 4:** Meta-analysis of PPV of different ML/AI models in assessing in-hospital mortality among AKI patients.

Model	No. of studies; no. of cohorts	No. of mortality/sample size	Pooled PPV (95% CI)	I^2^ (95% CI); *P*-values	Egger's test (*P*-value)
BLS models	1 study; 2 D and 2 V cohort	278/540	0.768 (0.628–0.909)	90.01% (77.34%–95.59%); *P *< .0001	0.2099
ANN/MLP	1 study; 1 D and 1 V cohort	962/3201	0.585 (0.515–0.654)	68.02% (0.00%–92.78%); *P *= .0770	<0.0001
RF	3 studies; 4 D and 4 V cohort	3071/13 328	0.568 (0.429–0.708)	99.80% (99.76%–99.83%); *P *< .0001	0.0272
XGBoost	2 studies; 2 D and 2 V cohort	2793/12 788	0.44 (0.266–0.613)	99.89% (99.87%–99.91%); *P *< .0001	0.6821
SVM	2 studies; 2 D and 2 V cohort	2793/12 788	0.444 (0.277–0.611)	99.78% (99.71%–99.82%); *P *< .0001	0.5386
Logistic regression	2 studies; 2 D and 2 V cohort	2793/12 788	0.43 (0.260–0.600)	99.88% (99.86%–99.90%); *P *< .0001	0.1635
PCM	1 study; 1 D and 1 V cohort	1831/9587	0.295 (0.069–0.520)	99.95% (99.94%–99.96%); *P *< .0001	<0.0001

Fixed effect models, for other random effect models.

a This clinical model was restricted to 14–15 variables that were top features of different ML models and were deemed by the investigators as pragmatic given its routine use/access to in clinical practice.

D: derivation, V: validation.

**Table 5:** Meta-analysis of NPV of different ML/AI models in assessing in-hospital mortality among AKI patients.

Model	No. of studies; no. of cohorts	No. of mortality/sample size	Pooled NPV (95% CI)	I^2^ (95% CI); *P*-values	Egger's test (*P*-value)
PCM^a^	1 study; 1 D and 1 V cohort	1831/9587	0.925 (0.876–0.974)	99.92% (99.89%–99.94%); *P *< .0001	<.0001
XGBoost	2 studies; 2 D and 2 V cohort	2793/12 788	0.892 (0.860–0.924)	97.55% (95.78%–98.57%); *P *< .0001	.5683
SVM	2 studies; 2 D and 2 V cohort	2793/12 788	0.888 (0.845–0.932)	98.57% (97.73%–99.09%); *P *< .0001	.7656
Logistic regression	2 studies; 2 D and 2 V cohort	2793/12 788	0.881 (0.831–0.931)	99.88% (99.86%–99.90%); *P *< .0001	.8033
RF	3 studies; 4 D and 4 V cohort	3071/13 328	0.858 (0.826–0.890)	99.49% (99.37%–99.59%); *P *< .0001	.5362
ANN/MLP	1 study; 1 D and 1 V cohort	962/3201	0.834 (0.782–0.885)	83.48% (31.43%–96.02%); *P *= .0139	<.0001
BLS models	1 study; 2 D and 2 V cohort	278/540	0.762 (0.657–0.867)	74.85% (30.15%–90.95%); *P *= .0076	.1319

a This clinical model was restricted to 14–15 variables that were top features of different ML models and were deemed by the investigators as pragmatic given its routine use/access to in clinical practice.

D: derivation; V: validation.

**Table 6:** Meta-analysis of accuracy of different ML/AI models in assessing in-hospital mortality among AKI patients.

Model	No. of studies; no. of cohorts	No. of mortality/sample size	Pooled accuracy (95% CI)	I^2^ (95% CI); *P*-values	Egger's test (*P*-value)
BLS models^a^	1 study; 2 D and 2 V cohort	278/540	0.742 (0.706–0.778)	19.54% (0.00%–89.61%); *P *= .2923	.1512
RF	3 studies; 4 D and 3 V cohort	4699/29 171	0.712 (0.680–0.745)	96.3% (94.25%–97.62%); *P *< .0001	.7677
SVM	2 studies; 2 D and 1 V cohort	4421/28 631	0.697 (0.656–0.738)	96.92% (93.75%–98.48%); *P *< .0001	.5825
PCM^b^	1 study; 1 D and 1 V cohort	1831/9587	0.680 (0.621–0.739)	99.28% (98.66%–99.61%); *P *< .0001	<.0001
Logistic regression	1 study; 1 D and 1 V cohort	1831/9587	0.681 (0.602–0.759)	98.42% (96.51%–99.29%); *P *< .0001	<.0001
XGBoost	1 study; 1 D and 1 V cohort	1831/9587	0.666 (0.578–0.754)	97.22% (92.90%–98.91%); *P *< .0001	<.0001
ANN/MLP	1 study; 1 D	2590/19 044	0.666 (0.626–0.705)	No meta-analysis done	
SAPS II	1 study; 1 D	2590/19 044	0.580 (0.538–0.621)	No meta-analysis done	

a Fixed effect models, for other random effect models.

b This clinical model was restricted to 14–15 variables that were top features of different ML models and were deemed by the investigators as pragmatic given its routine use/access to in clinical practice.

No meta-analysis done as there was only one cohort.

D: derivation; V: validation.

**Table 7a:** Meta-analysis of the AUC for the logistic regression model across different studies in assessing in-hospital mortality among AKI patients.

Study	No. of mortality	Sample size	AUC (95% CI)	Random weight (%)
Li *et al.* 2022 (D)	790	2666	0.812 (0.792–0.832)	12.85
Li *et al.* 2022 (V)	172	535	0.739 (0.692–0.786)	11.98
Neyra *et al.* 2022 (D)	1610	7354	0.770 (0.750–0.790)	12.85
Neyra *et al.* 2022 (V)	221	2233	0.770 (0.760–0.780)	13.00
Chen *et al.* 2021 (D)	32	196	0.947 (0.896–0.998)	11.81
Chen *et al.* 2021 (V)	11	52	0.971 (0.942–1.000)	12.61
Saly *et al.* 2017 (D)	108	1098	0.900 (0.871–0.929)	12.61
Saly *et al.* 2017 (V)	112	1143	0.800 (0.761–0.839)	12.29
Total (random effects)	3056	15 277	0.838 (0.781–0.895)	100.00

D: derivation; V: validation.

**Table 7b:** Meta-analysis of the AUC for the RF model across different studies in assessing in-hospital mortality among AKI patients.

Study	No. of mortality	Sample size	AUC (95% CI)	Random weight (%)
Li *et al.* 2022 (D)	790	2666	0.855 (0.837–0.873)	8.13
Li *et al.* 2022 (V)	172	535	0.689 (0.638–0.740)	6.85
Neyra *et al.* 2022 (D)	1610	7354	0.79 (0.780–0.800)	8.27
Neyra *et al.* 2022 (V)	221	2233	0.74 (0.738–0.742)	8.34
Zha *et al.* 2022 (D)	97	189	0.86 (0.809–0.911)	6.85
Zha *et al.* 2022 (D)	97	189	0.85 (0.799–0.901)	6.85
Zha *et al.* 2022 (V)	42	81	0.78 (0.680–0.880)	4.54
Zha *et al.* 2022 (V)	42	81	0.72 (0.669–0.771)	6.85
Ponce *et al.* 2021 (D)	436	697	0.894 (0.859–0.929)	7.55
Ponce *et al.* 2021 (V)	108	173	0.831 (0.772–0.890)	6.46
Kang *et al.* 2020	1019	1571	0.768 (0.727–0.809)	7.3
Lin *et al.* 2019	2590	19 044	0.866 (0.862–0.870)	8.33
Saly *et al.* 2017 (D)	108	1098	0.85 (0.799–0.901)	6.85
Saly *et al.* 2017 (V)	112	1143	0.8 (0.749–0.851)	6.85
Total (random effects)	7444	37 054	0.808 (0.776–0.839)	100

D: derivation; V: validation.

**Table 7c:** Meta-analysis of the AUC for the BLS model across different studies in assessing in-hospital mortality among AKI patients.

Study	No. of mortality	Sample size	AUC (95% CI)	Fixed weight (%)
Zha *et al.* 2022 (D)	97	189	0.870 (0.819–0.921)	38.77
Zha *et al.* 2022 (D)	97	189	0.870 (0.819–0.921)	38.77
Zha *et al.* 2022 (V)	42	81	0.820 (0.730–0.910)	12.39
Zha *et al.* 2022 (V)	42	81	0.750 (0.650–0.850)	10.08
Total (fixed effects)	278	540	0.852 (0.820–0.883)	100

D: derivation; V: validation.

**Table 7d:** Meta-analysis of the AUC for the ENF fitted model across different studies in assessing in-hospital mortality among AKI patients.

Study	No. of mortality	Sample size	AUC (95% CI)	Fixed weight (%)
Ponce *et al.* 2021 (D)	436	697	0.877 (0.824–0.930)	55.25
Ponce *et al.* 2021 (V)	108	173	0.821 (0.762–0.880)	44.75
Total (fixed effects)	544	870	0.852 (0.813–0.891)	100

D: derivation; V: validation.

**Table 7e:** Meta-analysis of the AUC for the XGBoost model across different studies in assessing in-hospital mortality among AKI patients.

Study	No. of mortality	Sample size	AUC (95% CI)	Random weight (%)
Li *et al.* 2022 (D)	790	2666	0.899 (0.883–0.915)	15.33
Li *et al.* 2022 (V)	172	535	0.716 (0.667–0.765)	13.79
Neyra *et al.* 2022 (D)	1610	7354	0.78 (0.778–0.782)	15.53
Neyra *et al.* 2022 (V)	221	2233	0.7 (0.680–0.720)	15.22
Ponce *et al.* 2021 (D)	436	697	0.886 (0.821–0.951)	12.72
Ponce *et al.* 2021 (V)	108	173	0.823 (0.766–0.880)	13.27
Kang *et al.* 2020	1019	1571	0.754 (0.711–0.797)	14.14
Total (random effects)	4356	15 229	0.793 (0.738–0.847)	100

D: derivation; V: validation.

**Table 7f:** Meta-analysis of the AUC for the SVM model across different studies in assessing in-hospital mortality among AKI patients.

Study	No. of mortality	Sample size	AUC (95% CI)	Random weight (%)
Li *et al.* 2022 (D)	790	2666	0.823 (0.803–0.843)	17.38
Li *et al.* 2022 (V)	172	535	0.759 (0.714–0.804)	13.45
Neyra *et al.* 2022 (D)	1610	7354	0.77 (0.768–0.772)	18.64
Neyra *et al.* 2022 (V)	221	2233	0.78 (0.770–0.790)	18.32
Kang *et al.* 2020	1019	1571	0.755 (0.712–0.798)	13.78
Lin *et al.* 2019	2590	19 044	0.861 (0.853–0.869)	18.43
Total (random effects)	6402	33 403	0.794 (0.763–0.826)	100

D: derivation; V: validation.

**Table 7g:** Meta-analysis of the AUC for the ANN/MLP model across different studies in assessing in-hospital mortality among AKI patients.

Study	No. of mortality	Sample size	AUC (95% CI)	Random weight (%)
Li *et al.* 2022 (D)	790	2666	0.824 (0.804–0.844)	27.88
Li *et al.* 2022 (V)	172	535	0.731 (0.684–0.778)	21.27
Kang *et al.* 2020	1019	1571	0.762 (0.719–0.805)	22.29
Lin *et al.* 2019	2590	19 044	0.833 (0.817–0.849)	28.55
Total (random effects)	4571	23 816	0.793 (0.752–0.833)	100

D: derivation; V: validation.

**Table 7h:** Meta-analysis of the AUC for the PCM across different studies.

Study	No. of mortality	Sample size	AUC (95% CI)	Random weight (%)
Neyra *et al.* 2022 (D)	1610	7354	0.79 (0.780–0.800)	49.04
Neyra *et al.* 2022 (V)	221	2233	0.74 (0.738–0.742)	50.96
Total (random effects)	1831	9587	0.765 (0.716–0.814)	100

D: derivation; V: validation.

### Sensitivity/recall, specificity, PPV and NPV

A total of four studies reported the data on these outcome measures for seven different ML/AI models.

**Figure 2:**
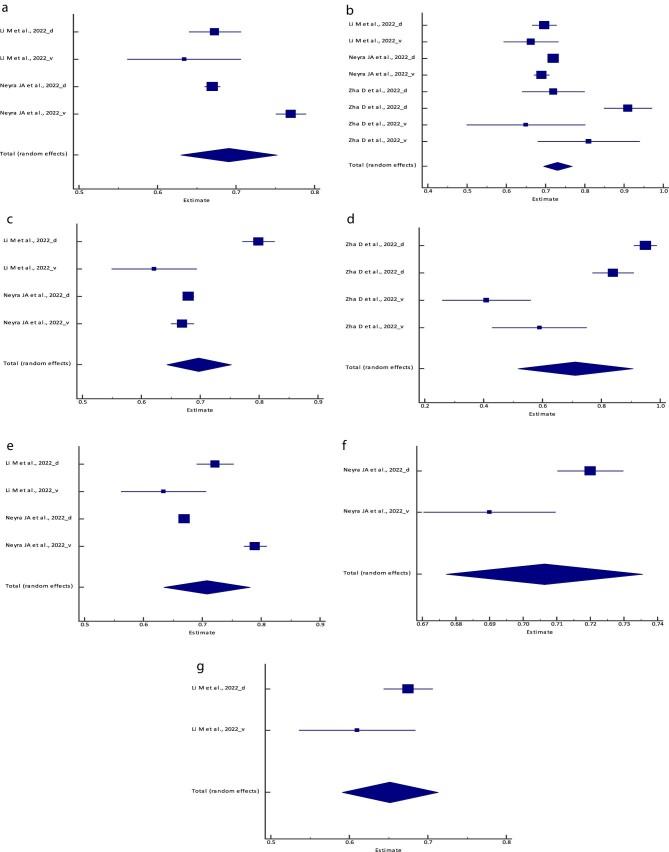
Forest plot of the meta-analysis of (**a**) the logistic regression model, (**b**) the RF model, (**c**) the XGBoost model, (**d**) the BLS model, (**e**) the SVM model, (**f**) the PCM and (**g**) the ANN/MLP model sensitivity across different studies. The lower diamond in each graph represents the pooled estimate sensitivity.

**Figure 3:**
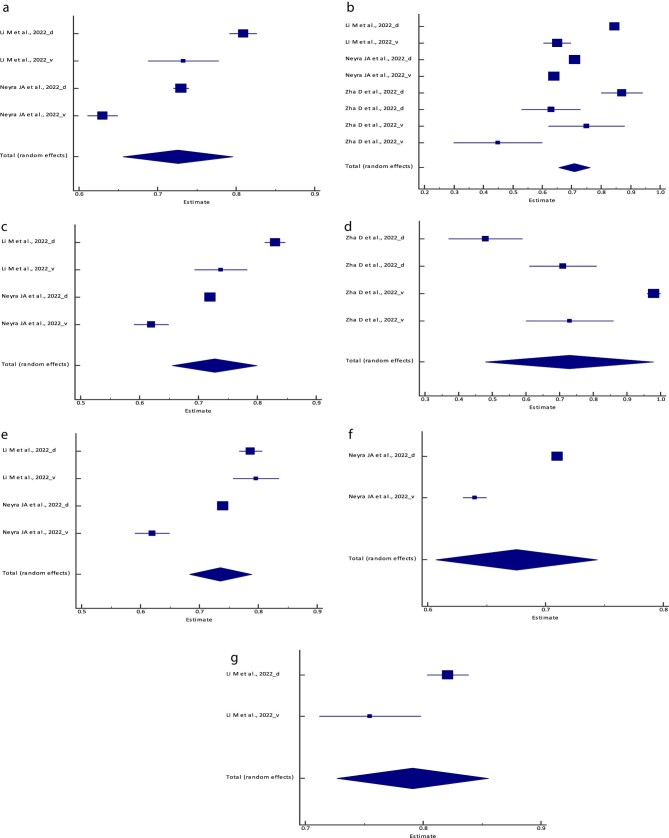
Forest plot of the meta-analysis of (**a**) the logistic regression model, (**b**) the RF model, (**c**) the XGBoost model, (**d**) the BLS model, (**e**) the SVM model, (**f**) the PCM and (**g**) the ANN/MLP model specificity across different studies. The lower diamond in each graph represents the pooled estimate specificity.

**Figure 4:**
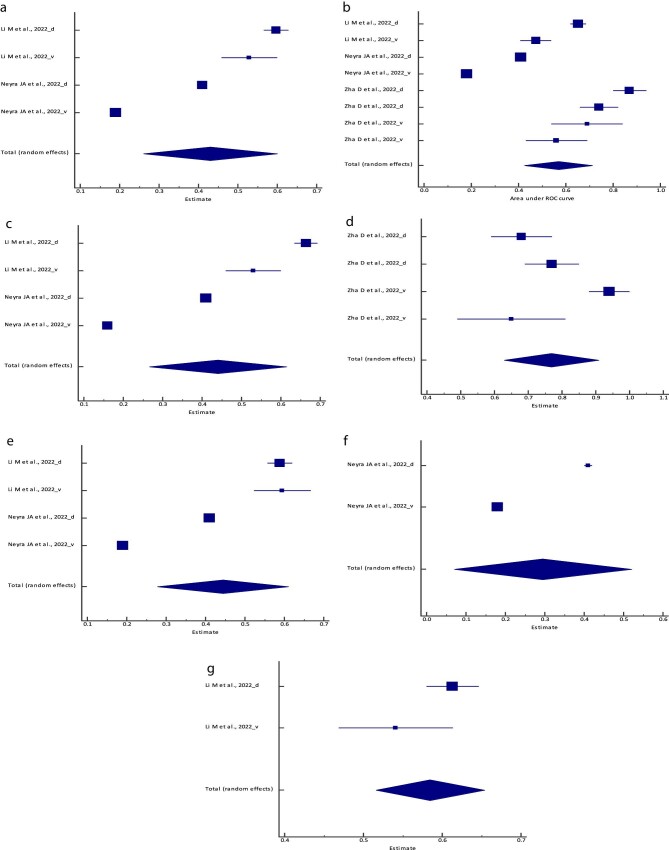
Forest plot of the meta-analysis of (**a**) the logistic regression model, (**b**) the RF model, (**c**) the XGBoost, (**d**) the BLS model, (**e**) the SVM model, (**f**) the PCM and (**g**) the ANN/MLP model PPV across different studies. The lower diamond in each graph represents the pooled estimate PPV.

**Figure 5:**
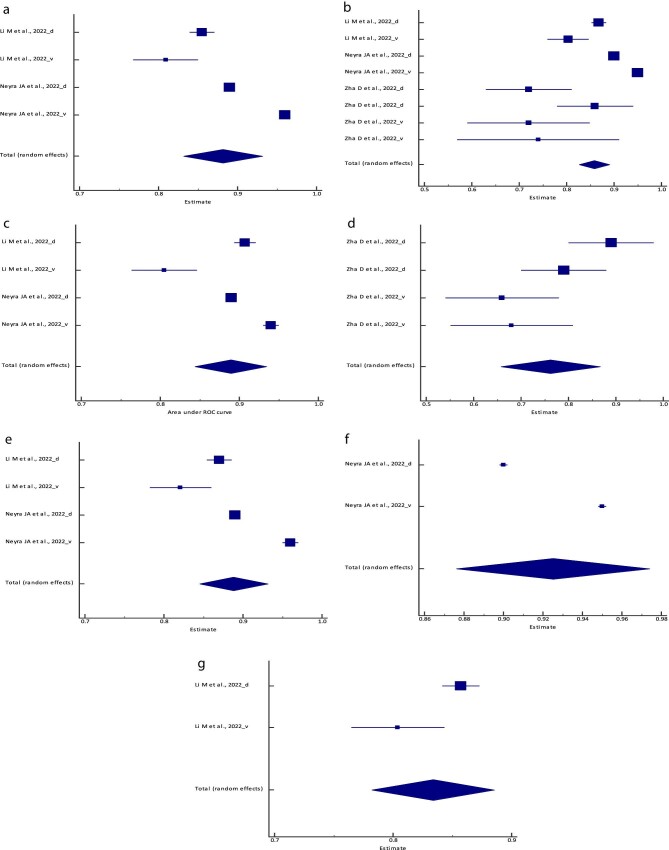
Forest plot of the meta-analysis of (**a**) the logistic regression model, (**b**) the RF model, (**c**) the XGBoost model, (**d**) the BLS model, (**e**) the SVM model, (**f**) the PCM and (**g**) the ANN/MLP model NPV across different studies. The lower diamond in each graph represents the pooled estimate NPV.

The pooled (95% CI) sensitivity was observed to be highest for RF model [0.73 (0.694–0.767)] and lowest for ANN/MLP [0.652 (0.591–0.713)] as shown in Table 2. The pooled (95% CI) specificity was observed to be highest for ANN/MLP [0.791 (0.727–0.855)] and lowest for PCM [0.675 (0.607–0.744)] as shown in Table 3. The pooled (95% CI) PPV was observed to be highest for BLS models [0.768 (0.628–0.909)] and lowest for PCM [0.295 (0.069–0.520)] (Table 4). The pooled (95% CI) NPV was observed to be highest for PCM [0.925 (0.876–0.974)] and lowest for BLS models [0.762 (0.657–0.867)] (Table 5). There was no evidence of publication bias for most of the models based on Egger's test (*P *> .05). Tables 8a–8g to 11a–11g and Figs 2–5 provide meta-analysis of sensitivity, specificity, PPV and NPV for individual ML/AI models across different studies.

**Table 8a:** Meta-analysis of sensitivity for the logistic regression model across different studies in assessing in-hospital mortality among AKI patients.

Study	No. of mortality	Sample size	Sensitivity (95% CI)	Random weight (%)
Li *et al.* 2022 (D)	790	2666	0.673 (0.640–0.706)	25.6
Li *et al.* 2022 (V)	172	535	0.634 (0.561–0.707)	19.98
Neyra *et al.* 2022 (D)	1610	7354	0.67 (0.660–0.680)	27.49
Neyra *et al.* 2022 (V)	221	2233	0.77 (0.750–0.790)	26.93
Total (random effects)	2793	12 788	0.691 (0.629–0.752)	100

D: derivation; V: validation.

**Table 8b:** Meta-analysis of sensitivity for the RF model across different studies in assessing in-hospital mortality among AKI patients.

Study	No. of mortality	Sample size	Sensitivity (95% CI)	Random weight (%)
Li *et al.* 2022 (D)	790	2666	0.697 (0.666–0.728)	17.29
Li *et al.* 2022 (V)	172	535	0.663 (0.592–0.734)	11.36
Neyra *et al.* 2022 (D)	1610	7354	0.720 (0.710–0.730)	19.56
Neyra *et al.* 2022 (V)	221	2233	0.690 (0.670–0.710)	18.76
Zha *et al.* 2022 (D)	97	189	0.720 (0.640–0.800)	10.08
Zha *et al.* 2022 (D)	97	189	0.910 (0.849–0.971)	12.78
Zha *et al.* 2022 (V)	42	81	0.650 (0.499–0.801)	4.5
Zha *et al.* 2022 (V)	42	81	0.810 (0.681–0.939)	5.66
Total (random effects)	3071	13 328	0.730 (0.694–0.767)	100

D: derivation; V: validation.

**Table 8c:** Meta-analysis of sensitivity for the XGBoost model across different studies in assessing in-hospital mortality among AKI patients.

Study	No. of mortality	Sample size	Sensitivity (95% CI)	Random weight (%)
Li *et al.* 2022 (D)	790	2666	0.799 (0.772–0.826)	26.24
Li *et al.* 2022 (V)	172	535	0.622 (0.549–0.695)	18.79
Neyra *et al.* 2022 (D)	1610	7354	0.68 (0.670–0.690)	27.85
Neyra *et al.* 2022 (V)	221	2233	0.67 (0.650–0.690)	27.12
Total (random effects)	2793	12 788	0.698 (0.643–0.752)	100

D: derivation; V: validation.

**Table 8d:** Meta-analysis of sensitivity for the BLS model across different studies in assessing in-hospital mortality among AKI patients.

Study	No. of mortality	Sample size	Sensitivity (95% CI)	Random weight (%)
Zha *et al.* 2022 (D)	97	189	0.95 (0.911–0.989)	27.05
Zha *et al.* 2022 (D)	97	189	0.84 (0.769–0.911)	26.4
Zha *et al.* 2022 (V)	42	81	0.41 (0.259–0.561)	23.49
Zha *et al.* 2022 (V)	42	81	0.59 (0.429–0.751)	23.05
Total (random effects)	278	540	0.711 (0.516–0.906)	100

D: derivation; V: validation.

**Table 8e:** Meta-analysis of sensitivity for the SVM model across different studies in assessing in-hospital mortality among AKI patients.

Study	No. of mortality	Sample size	Sensitivity (95% CI)	Random weight (%)
Li *et al.* 2022 (D)	790	2666	0.722 (0.691–0.753)	25.58
Li *et al.* 2022 (V)	172	535	0.634 (0.561–0.707)	21.25
Neyra *et al.* 2022 (D)	1610	7354	0.67 (0.668–0.672)	26.83
Neyra *et al.* 2022 (V)	221	2233	0.79 (0.770–0.810)	26.33
Total (random effects)	2793	12 788	0.707 (0.634–0.781)	100

D: derivation; V: validation.

**Table 8f:** Meta-analysis of sensitivity for the PCM across different studies in assessing in-hospital mortality among AKI patients.

Study	No. of mortality	Sample size	Sensitivity (95% CI)	Random weight (%)
Neyra *et al.* 2022 (D)	1610	7354	0.72 (0.710–0.730)	54.17
Neyra *et al.* 2022 (V)	221	2233	0.69 (0.670–0.710)	45.83
Total (random effects)	1831	9587	0.706 (0.677–0.736)	100

D: derivation; V: validation.

**Table 8g:** Meta-analysis of sensitivity for the ANN/MLP model across different studies in assessing in-hospital mortality among AKI patients.

Study	No. of mortality	Sample size	Sensitivity (95% CI)	Random weight (%)
Li *et al.* 2022 (D)	790	2666	0.675 (0.644–0.706)	64.06
Li *et al.* 2022 (V)	172	535	0.610 (0.536–0.684)	35.94
Total (random effects)	962	3201	0.652 (0.591–0.713)	100

D: derivation; V: validation.

**Table 9a:** Meta-analysis of specificity for the logistic regression model across different studies in assessing in-hospital mortality among AKI patients.

Study	No. of mortality	Sample size	Specificity (95% CI)	Random weight (%)
Li *et al.* 2022 (D)	790	2666	0.809 (0.791–0.827)	25.48
Li *et al.* 2022 (V)	172	535	0.733 (0.688–0.778)	23.38
Neyra *et al.* 2022 (D)	1610	7354	0.73 (0.720–0.740)	25.76
Neyra *et al.* 2022 (V)	221	2233	0.63 (0.610–0.650)	25.38
Total (random effects)	2793	12 788	0.725 (0.655–0.795)	100

D: derivation; V: validation.

**Table 9b:** Meta-analysis of specificity for the RF model across different studies in assessing in-hospital mortality among AKI patients.

Study	No. of mortality	Sample size	Specificity (95% CI)	Random weight (%)
Li *et al.* 2022 (D)	790	2666	0.845 (0.829–0.861)	15.83
Li *et al.* 2022 (V)	172	535	0.651 (0.604–0.698)	14.25
Neyra *et al.* 2022 (D)	1610	7354	0.71 (0.708–0.712)	16.05
Neyra *et al.* 2022 (V)	221	2233	0.64 (0.630–0.650)	15.97
Zha *et al.* 2022 (D)	97	189	0.87 (0.799–0.941)	12.5
Zha *et al.* 2022 (D)	97	189	0.63 (0.530–0.730)	10.22
Zha *et al.* 2022 (V)	42	81	0.75 (0.621–0.879)	8.2
Zha *et al.* 2022 (V)	42	81	0.45 (0.299–0.601)	6.97
Total (random effects)	3071	13 328	0.709 (0.656–0.762)	100

D: derivation; V: validation.

**Table 9c:** Meta-analysis of specificity for the XGBoost model across different studies in assessing in-hospital mortality among AKI patients.

Study	No. of mortality	Sample size	Specificity (95% CI)	Random weight (%)
Li *et al.* 2022 (D)	790	2666	0.83 (0.812–0.848)	25.57
Li *et al.* 2022 (V)	172	535	0.738 (0.693–0.783)	23.56
Neyra *et al.* 2022 (D)	1610	7354	0.72 (0.718–0.722)	25.97
Neyra *et al.* 2022 (V)	221	2233	0.62 (0.591–0.649)	24.89
Total (random effects)	2793	12 788	0.727 (0.656–0.799)	100

D: derivation; V: validation.

**Table 9d:** Meta-analysis of specificity for the BLS model across different studies in assessing in-hospital mortality among AKI patients.

Study	No. of mortality	Sample size	Specificity (95% CI)	Random weight (%)
Zha *et al.* 2022 (D)	97	189	0.48 (0.370–0.590)	24.76
Zha *et al.* 2022 (D)	97	189	0.71 (0.610–0.810)	24.97
Zha *et al.* 2022 (V)	42	81	0.98 (0.960–1.000)	25.96
Zha *et al.* 2022 (V)	42	81	0.73 (0.601–0.859)	24.31
Total (random effects)	278	540	0.728 (0.478–0.978)	100

D: derivation; V: validation.

**Table 9e:** Meta-analysis of specificity for the SVM model across different studies in assessing in-hospital mortality among AKI patients.

Study	No. of mortality	Sample size	Specificity (95% CI)	Random weight (%)
Li *et al.* 2022 (D)	790	2666	0.787 (0.767–0.807)	25.66
Li *et al.* 2022 (V)	172	535	0.796 (0.757–0.835)	23.19
Neyra *et al.* 2022 (D)	1610	7354	0.74 (0.738–0.742)	26.59
Neyra *et al.* 2022 (V)	221	2233	0.62 (0.591–0.649)	24.57
Total (random effects)	2793	12 788	0.736 (0.683–0.788)	100

D: derivation; V: validation.

**Table 9f:** Meta-analysis of specificity for the PCM across different studies in assessing in-hospital mortality among AKI patients.

Study	No. of mortality	Sample size	Specificity (95% CI)	Random weight (%)
Neyra *et al.* 2022 (D)	1610	7354	0.71 (0.708–0.712)	50.24
Neyra *et al.* 2022 (V)	221	2233	0.64 (0.630–0.650)	49.76
Total (random effects)	1831	9587	0.675 (0.607–0.744)	100

D: derivation; V: validation.

**Table 9g:** Meta-analysis of specificity for the ANN/MLP model across different studies in assessing in-hospital mortality among AKI patients.

Study	No. of mortality	Sample size	Specificity (95% CI)	Random weight (%)
Li *et al.* 2022 (D)	790	2666	0.821 (0.803–0.839)	54.63
Li *et al.* 2022 (V)	172	535	0.755 (0.712–0.798)	45.37
Total (random effects)	962	3201	0.791 (0.727–0.855)	100

D: derivation; V: validation.

**Table 10a:** Meta-analysis of PPV for the logistic regression model across different studies in assessing in-hospital mortality among AKI patients.

Study	No. of mortality	Sample size	PPV (95% CI)	Random weight (%)
Li *et al.* 2022 (D)	790	2666	0.597 (0.566–0.628)	25.11
Li *et al.* 2022 (V)	172	535	0.529 (0.458–0.600)	24.27
Neyra *et al.* 2022 (D)	1610	7354	0.41 (0.400–0.420)	25.3
Neyra *et al.* 2022 (V)	221	2233	0.19 (0.188–0.192)	25.32
Total (random effects)	2793	12 788	0.43 (0.260–0.600)	100

D: derivation; V: validation.

**Table 10b:** Meta-analysis of PPV for the RF model across different studies in assessing in-hospital mortality among AKI patients.

Study	No. of mortality	Sample size	PPV (95% CI)	Random weight (%)
Li *et al.* 2022 (D)	790	2666	0.653 (0.620–0.686)	12.94
Li *et al.* 2022 (V)	172	535	0.473 (0.408–0.538)	12.69
Neyra *et al.* 2022 (D)	1610	7354	0.41 (0.400–0.420)	13.02
Neyra *et al.* 2022 (V)	221	2233	0.18 (0.178–0.182)	13.03
Zha *et al.* 2022 (D)	97	189	0.87 (0.799–0.941)	12.63
Zha *et al.* 2022 (D)	97	189	0.74 (0.660–0.820)	12.52
Zha *et al.* 2022 (V)	42	81	0.69 (0.539–0.841)	11.39
Zha *et al.* 2022 (V)	42	81	0.56 (0.431–0.689)	11.78
Total (random effects)	3071	13 328	0.568 (0.425–0.712)	100

D: derivation; V: validation.

**Table 10c:** Meta-analysis of PPV for the XGBoost model across different studies in assessing in-hospital mortality among AKI patients.

Study	No. of mortality	Sample size	PPV (95% CI)	Random weight (%)
Li *et al.* 2022 (D)	790	2666	0.664 (0.635–0.693)	25.12
Li *et al.* 2022 (V)	172	535	0.53 (0.459–0.601)	24.3
Neyra *et al.* 2022 (D)	1610	7354	0.41 (0.408–0.412)	25.3
Neyra *et al.* 2022 (V)	221	2233	0.16 (0.150–0.170)	25.28
Total (random effects)	2793	12 788	0.44 (0.265–0.614)	100

D: derivation; V: validation.

**Table 10d:** Meta-analysis of PPV for the BLS model across different studies in assessing in-hospital mortality among AKI patients.

Study	No. of mortality	Sample size	PPV (95% CI)	Random weight (%)
Zha *et al.* 2022 (D)	97	189	0.68 (0.590–0.770)	25.67
Zha *et al.* 2022 (D)	97	189	0.77 (0.690–0.850)	26.24
Zha *et al.* 2022 (V)	42	81	0.94 (0.879–1.001)	27.24
Zha *et al.* 2022 (V)	42	81	0.65 (0.489–0.811)	20.86
Total (random effects)	278	540	0.768 (0.628–0.909)	100

D: derivation; V: validation.

**Table 10e:** Meta-analysis of PPV for the SVM model across different studies in assessing in-hospital mortality among AKI patients.

Study	No. of mortality	Sample size	PPV (95% CI)	Random weight (%)
Li *et al.* 2022 (D)	790	2666	0.588 (0.557–0.619)	25.13
Li *et al.* 2022 (V)	172	535	0.595 (0.522–0.668)	24.2
Neyra *et al.* 2022 (D)	1610	7354	0.41 (0.400–0.420)	25.34
Neyra *et al.* 2022 (V)	221	2233	0.19 (0.180–0.200)	25.34
Total (random effects)	2793	12 788	0.444 (0.277–0.611)	100

D: derivation; V: validation.

**Table 10f:** Meta-analysis of PPV for the PCM across different studies in assessing in-hospital mortality among AKI patients.

Study	No. of mortality	Sample size	PPV (95% CI)	Random weight (%)
Neyra *et al.* 2022 (D)	1610	7354	0.41 (0.400–0.420)	49.98
Neyra *et al.* 2022 (V)	221	2233	0.18 (0.178–0.182)	50.02
Total (random effects)	1831	9587	0.295 (0.069–0.520)	100

D: derivation; V: validation.

**Table 10g:** Meta-analysis of PPV for the ANM/MLP model across different studies in assessing in-hospital mortality among AKI patients.

Study	No. of mortality	Sample size	PPV (95% CI)	Random weight (%)
Li *et al.* 2022 (D)	790	2666	0.613 (0.580–0.646)	60.42
Li *et al.* 2022 (V)	172	535	0.541 (0.468–0.614)	39.58
Total (random effects)	962	3201	0.585 (0.515–0.654)	100

D: derivation; V: validation.

**Table 11a:** Meta-analysis of NPV for the logistic regression model across different studies in assessing in-hospital mortality among AKI patients.

Study	No. of mortality	Sample size	NPV (95% CI)	Random weight (%)
Li *et al.* 2022 (D)	790	2666	0.855 (0.839–0.871)	25.5
Li *et al.* 2022 (V)	172	535	0.809 (0.768–0.850)	22.21
Neyra *et al.* 2022 (D)	1610	7354	0.89 (0.888–0.892)	26.14
Neyra *et al.* 2022 (V)	221	2233	0.96 (0.958–0.962)	26.14
Total (random effects)	2793	12 788	0.881 (0.831–0.931)	100

D: derivation; V: validation.

**Table 11b:** Meta-analysis of NPV for the RF model across different studies in assessing in-hospital mortality among AKI patients.

Study	No. of mortality	Sample size	NPV (95% CI)	Random weight (%)
Li *et al.* 2022 (D)	790	2666	0.868 (0.852–0.884)	19.5
Li *et al.* 2022 (V)	172	535	0.803 (0.760–0.846)	14.97
Neyra *et al.* 2022 (D)	1610	7354	0.9 (0.898–0.902)	20.43
Neyra *et al.* 2022 (V)	221	2233	0.95 (0.948–0.952)	20.43
Zha *et al.* 2022 (D)	97	189	0.72 (0.630–0.810)	7.86
Zha *et al.* 2022 (D)	97	189	0.86 (0.780–0.940)	9
Zha *et al.* 2022 (V)	42	81	0.72 (0.591–0.849)	4.76
Zha *et al.* 2022 (V)	42	81	0.74 (0.569–0.911)	3.04
Total (random effects)	3071	13 328	0.858 (0.826–0.890)	100

D: derivation; V: validation.

**Table 11c:** Meta-analysis of NPV for the XGBoost model across different studies in assessing in-hospital mortality among AKI patients.

Study	No. of mortality	Sample size	NPV (95% CI)	Random weight (%)
Li *et al.* 2022 (D)	790	2666	0.907 (0.893–0.921)	25.8
Li *et al.* 2022 (V)	172	535	0.805 (0.764–0.846)	21.67
Neyra *et al.* 2022 (D)	1610	7354	0.89 (0.888–0.892)	26.42
Neyra *et al.* 2022 (V)	221	2233	0.94 (0.930–0.950)	26.11
Total (random effects)	2793	12 788	0.889 (0.844–0.934)	100

D: derivation; V: validation.

**Table 11d:** Meta-analysis of NPV for the BLS model across different studies in assessing in-hospital mortality among AKI patients.

Study	No. of mortality	Sample size	NPV (95% CI)	Random weight (%)
Zha *et al.* 2022 (D)	97	189	0.89 (0.800–0.980)	27.06
Zha *et al.* 2022 (D)	97	189	0.79 (0.700–0.880)	27.06
Zha *et al.* 2022 (V)	42	81	0.66 (0.540–0.780)	23.52
Zha *et al.* 2022 (V)	42	81	0.68 (0.551–0.809)	22.36
Total (random effects)	278	540	0.762 (0.657–0.867)	100

D: derivation; V: validation.

**Table 11e:** Meta-analysis of NPV for the SVM model across different studies in assessing in-hospital mortality among AKI patients.

Study	No. of mortality	Sample size	NPV (95% CI)	Random weight (%)
Li *et al.* 2022 (D)	790	2666	0.87 (0.854–0.886)	25.6
Li *et al.* 2022 (V)	172	535	0.821 (0.782–0.860)	21.78
Neyra *et al.* 2022 (D)	1610	7354	0.89 (0.888–0.892)	26.48
Neyra *et al.* 2022 (V)	221	2233	0.96 (0.950–0.970)	26.14
Total (random effects)	2793	12 788	0.888 (0.845–0.932)	100

D: derivation; V: validation.

**Table 11f:** Meta-analysis of NPV for the PCM across different studies in assessing in-hospital mortality among AKI patients.

Study	No. of mortality	Sample size	PPV (95% CI)	Random weight (%)
Neyra *et al.* 2022 (D)	1610	7354	0.9 (0.898–0.902)	50
Neyra *et al.* 2022 (V)	221	2233	0.95 (0.948–0.952)	50
Total (random effects)	1831	9587	0.925 (0.876–0.974)	100

D: derivation; V: validation.

**Table 11g:** Meta-analysis of NPV for the ANN/MLP model across different studies in assessing in-hospital mortality among AKI patients.

Study	No. of mortality	Sample size	NPV (95% CI)	Random weight (%)
Li *et al.* 2022 (D)	790	2666	0.857 (0.841–0.873)	55.98
Li *et al.* 2022 (V)	172	535	0.804 (0.765–0.843)	44.02
Total (random effects)	962	3201	0.834 (0.782–0.885)	100

D: derivation; V: validation.

### Accuracy

**Figure 6:**
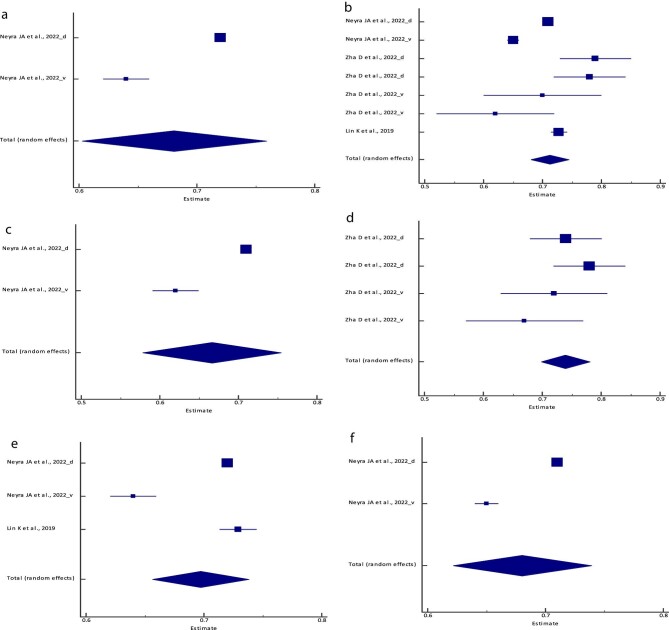
Forest plot of the meta-analysis of (**a**) the logistic regression model, (**b**) the RF model, (**c**) the XGBoost model, (**d**) the BLS model, (**e**) the SVM model and (**f**) the PCM accuracy across different studies. The lower diamond in each graph represents the pooled estimate accuracy.

A total of four studies reported the data on accuracy for six different ML/AI models. A meta-analysis was conducted for six models with data from one or more cohort. Across the models with meta-analysis, the pooled (95% CI) AUC was observed to be highest for BLS models [0.742 (0.706–0.778)] and lowest for XGBoost [0.666 (0.578–0.754)] (Table 6). Even considering the models with only one cohort, the accuracy for BLS models was observed to be the highest. There was no evidence of publication bias for BLS models, RF and SVM based on Egger's test (*P *> .05), but evidence of publication bias was observed for the other models. Tables 12a–12f and Fig. 6 provide meta-analysis results of accuracy for individual ML/AI models across different studies.

**Table 12a:** Meta-analysis of accuracy for the logistic regression model across different studies in assessing in-hospital mortality among AKI patients.

Study	No. of mortality	Sample size	Accuracy (95% CI)	Random weight (%)
Neyra *et al.* 2022 (D)	1610	7354	0.72 (0.718–0.722)	50.77
Neyra *et al.* 2022 (V)	221	2233	0.64 (0.620–0.660)	49.23
Total (random effects)	1831	9587	0.681 (0.602–0.759)	100

D: derivation; V: validation.

**Table 12b:** Meta-analysis of accuracy for the RF model across different studies in assessing in-hospital mortality among AKI patients.

Study	No. of mortality	Sample size	Accuracy (95% CI)	Random weight (%)
Neyra *et al.* 2022 (D)	1610	7354	0.71 (0.708–0.712)	21.02
Neyra *et al.* 2022 (V)	221	2233	0.65 (0.640–0.660)	20.64
Zha *et al.* 2022 (D)	97	189	0.79 (0.729–0.851)	12.06
Zha *et al.* 2022 (D)	97	189	0.78 (0.719–0.841)	12.06
Zha *et al.* 2022 (V)	42	81	0.7 (0.600–0.800)	6.98
Zha *et al.* 2022 (V)	42	81	0.62 (0.520–0.720)	6.98
Lin *et al.* 2019	2590	19 044	0.728 (0.714–0.742)	20.27
Total (random effects)	4699	29 171	0.712 (0.680–0.745)	100

D: derivation; V: validation.

**Table 12c:** Meta-analysis of accuracy for the XGBoost model across different studies in assessing in-hospital mortality among AKI patients.

Study	No. of mortality	Sample size	Accuracy (95% CI)	Random weight (%)
Neyra *et al.* 2022 (D)	1610	7354	0.71 (0.708–0.712)	51.38
Neyra *et al.* 2022 (V)	221	2233	0.62 (0.591–0.649)	48.62
Total (random effects)	1831	9587	0.666 (0.578–0.754)	100

D: derivation; V: validation.

**Table 12d:** Meta-analysis of accuracy for the BLS model across different studies in assessing in-hospital mortality among AKI patients.

Study	No. of mortality	Sample size	Accuracy (95% CI)	Fixed weight (%)
Zha *et al.* 2022 (D)	97	189	0.74 (0.679–0.801)	35.42
Zha *et al.* 2022 (D)	97	189	0.78 (0.719–0.841)	35.42
Zha *et al.* 2022 (V)	42	81	0.72 (0.630–0.810)	16.08
Zha *et al.* 2022 (V)	42	81	0.67 (0.570–0.770)	13.09
Total (fixed effects)	278	540	0.742 (0.706–0.778)	100

D: derivation; V: validation.

**Table 12e:** Meta-analysis of accuracy for the SVM model across different studies in assessing in-hospital mortality among AKI patients.

Study	No. of mortality	Sample size	Accuracy (95% CI)	Random weight (%)
Neyra *et al*. 2022 (D)	1610	7354	0.72 (0.718–0.722)	34.74
Neyra *et al*. 2022 (V)	221	2233	0.64 (0.620–0.660)	32.19
Lin *et al*. 2019	2590	19 044	0.729 (0.713–0.745)	33.07
Total (random effects)	4421	28 631	0.697 (0.656–0.738)	100

D: derivation; V: validation.

**Table 12f:** Meta-analysis of accuracy for the PCM across different studies in assessing in-hospital mortality among AKI patients.

Study	No. of mortality	Sample size	Accuracy (95% CI)	Random weight (%)
Neyra *et al*. 2022 (D)	1610	7354	0.71 (0.708–0.712)	50.33
Neyra *et al*. 2022 (V)	221	2233	0.65 (0.640–0.660)	49.67
Total (random effects)	1831	9587	0.68 (0.621–0.739)	100

D: derivation; V: validation.

## DISCUSSION

Conventional scoring systems and regression-based models have shown limited performance, emphasizing the need for more encapsulating predictive models through the exploration of ML methods [6]. The necessity for improved, more accurate and actionable prediction models could lead to targeted interventions and better allocation of clinical resources [6].

The results of this study describe the differences of statistical performance between different forms of ML/AI models in predicting in-hospital mortality in patients with AKI. By understanding the performance characteristics of each ML/AI model, proper use can be attained in guiding clinical practice and future research. The use of specific performance metrics throughout each study can be attributed to the primary objective of the study. The AUC measures the model's capability in discriminating between positive versus negative cases. The majority of included studies provided the AUC as a comprehensive statistic to describe each model's performance. More specifically, other studies may have also described other performance metrics including PPV, NPV and accuracy. Based on the clinical context, each call for prediction necessitates different goals and projected actionable interventions. Researchers and clinicians should carefully apply the referred statistics of each ML/AI model to their correct, corresponding potential use.

The study evaluated 14 different ML/AI models to describe their performance. The “best model” depends on various clinical factors, different goals, computational resources, dataset characteristics, model training databases and interpretability requirements. The performance statistics indicate that each ML/AI model exhibited diverse performance outcomes, particularly in relation to the prediction of hospital mortality. Among the evaluated models, the BLS model and ENF model had the highest AUC [0.852 (0.820–0.883) and 0.852 (0.813–0.891), respectively]. This indicates a reliable discriminatory model only in relation to the PCM when predicting hospital mortality in AKI patients. The PCM exhibited the highest NPV, a critical attribute in clinical decision-making. Despite its potential for occasional false positive signals, this model's strength as a “rule-out” tool makes it valuable in situations such as initial patient triage, allowing for further confirmatory re-evaluation when necessary. It is important to note that the BLS or ENF model did not show a statistically significant difference in AUC in comparison with the other listed ML/AI models. BLS/ENF models are therefore comparatively reliable in predicting in-hospital mortality in AKI patients when compared with the other referred ML/AI models. Although these models have been widely used in medical research and have been shown to perform well in predicting clinical outcomes, recent advances in ML models are able to improve prediction and efficiently handle large and complex datasets. The similar discriminatory efficiency of certain traditional ML models including logistic regression model, RF and XGBoost has been exhibited in a previous study as quantitatively described by the AUC [9].

The strengths and weaknesses of the other performance metrics can be applicable to other corresponding clinical situations. The ML/AI models showed varied performance regarding sensitivity, specificity, PPV, NPV and accuracy. However, for the three common ML models (RF, logistic regression and XGBoost), the pooled sensitivity, specificity, PPV and NPV values were observed to be within the 95% CI of each other, indicating insignificant difference in performance across these models. This suggests the importance of understanding individual model performance for their corresponding clinical application. Overall, different ML/AI models have a myriad of strengths and limitations and therefore a comprehensive approach is necessary to choose the most efficient model.

In relation to standard clinical scoring tools such as the SOFA, MOSAIC, APACHE II and SAPS II, the goal of ML/AI models is to offer different avenues of reaching accurate predictability. Instead of relying solely on clinical parameters and physiological measurements such as in these clinical scoring tools, ML/AI models have the capability to incorporate complex variables from a wide array of large datasets. In relation to AUC, the three common ML models of RF, logistic regression and XGBoost scored higher in comparison with SOFA, MOSAIC and APACHE II. Further, these three scoring clinical models scored significantly lower in comparison with ENF, SVM and ANN. The ML/AI models additionally showed similar discriminatory efficiency in relation to SAPS II, with ENF and BLS showing significantly different AUC in relation to SAPS II. This suggests the variation of discriminatory power of the mentioned ML/AI models in relation to certain scoring systems as compared with others.

This study assessed the degree of heterogeneity using the I^2^ tests, with some models showing a high degree which could be attributed to differences in patient population, data sources or model configurations. More specifically, this may include the limited number of ML model studies, the variety of different algorithm training populations, the variety of algorithm training methods, missing or invalid data impacting model performance, feature selections and the lack of external validation for two studies. It is important to identify such heterogeneities before applying these results to real-world settings. Research bias was also assessed using Egger's test. Publication bias is present when relative majority of studies are published with positive or statistically significant results as compared with negative or non-significant results. This bias could then lead to an overestimation of model performances. Researchers should be cautious of such biases and carefully analyze data quality, model interpretation and generalizability in their assessment. Furthermore, traditional ML models such as RF, logistic regression and XGBoost were more commonly used as compared with deep learning models such as ANN/MLP. This leads to an over-representation bias, a limited comparison and a limited applicability to other domains. Additionally, two of the included studies did not include validation cohorts. In examining the performance of any predictive model on unseen data, a validation dataset is crucial to ensure a given model is making accurate predictions. Although a model trained and tested on the same dataset may still create generalizable predictions, the training accuracy of a model is a less reliable metric to determine its feasibility in a deployable environment, especially considering the risk of overfitting. However, it is crucial to acknowledge the prevalence of models that are trained and tested on the same data due to the limited availability of a substantial set of clean and accessible data in the medical field. Due to restrictions in data availability, studies without an external validation set constitute a considerable proportion of work regarding the use of AI in medicine; as such, some studies without validation datasets were also considered within the scope of this study while acknowledging the limitations that come alongside them. Future research should include more diverse ML models and different datasets for each model in order to assess robustness and generalizability.

The findings of this study provide a foundation for further research in the field of AKI mortality prediction using ML/AI models including both linear and non-linear ML methods as compared with the standard clinical tools. By validating and further assessing the performance of certain models in specific clinical settings, accurate prediction rates can increase and be more actionable. Efforts should also be made to identify biases and limitations that hinder the use of ML/AI models in terms of data quality, model interpretation, generalizability and implementation.

## Supplementary Material

sfae150_Supplemental_File

## Data Availability

All data generated or analyzed during this study are included in respective cited articles and its supplementary information files.

## PROSPERO REGISTRATION

CRD42023437982.
