# Correction to: Using artificial intelligence to predict mortality in AKI patients: a systematic review/meta-analysis

**DOI:** 10.1093/ckj/sfae223

**Published:** 2024-08-01

This is a correction to: Rupesh Raina, Raghav Shah, Paul Nemer, Jared Fehlmen, Lena Nemer, Ali Murra, Abhishek Tibrewal, Sidharth Kumar Sethi, Javier A Neyra, Jay Koyner, Using artificial intelligence to predict mortality in AKI patients: a systematic review/meta-analysis, Clinical Kidney Journal, Volume 17, Issue 6, June 2024, sfae150, https://doi.org/10.1093/ckj/sfae150

Several changes have been made to the originally published version of this manuscript.

In the Results section of the Abstract, the following sentences required correction:

‘Eight studies with 37 032 AKI patients were included, with a mean age of 65.3 years. The in-hospital mortality was 18.0% in the derivation and 15.8% in the validation cohorts.’

‘The pooled (95% CI) AUC of BLS and ENF did not differ significantly from other models except PCM [Delong's test *P* = .022].’

The corrected sentences read as follows:

‘Eight studies with 37 032 AKI patients were included, with a mean age of 65.1 years. The in-hospital mortality was observed to be 19.8%.’

‘The pooled (95% CI) AUC of BLS and ENF did not differ significantly from other models except PCM [Delong's test *P* = .013].’

In the Results section, under subsection heading ‘Area under the curve’, the following sentences required correction:

‘Across these eight models, the pooled (95% CI) AUC was observed to be highest for BLS models [0.852 (0.820–0.883)] and lowest for a proposed simplified clinical model [0.765 (0.716–0.814)].’

‘There was no evidence of publication bias for most of the models based on Egger's test (*P* > .05) except ANN/MLP and PCM.’

The corrected sentences read as follows:

‘Across these eight models, the pooled (95% CI) AUC was observed to be highest for BLS models [0.852 (0.820–0.833] and elastic net final (ENF) model [0.852 (0.813–0.891], and lowest for proposed clinic model (PCM) [0.765 (0.716–0.814)].’

‘There was no evidence of publication bias for most of the models based on Egger's test (*P* > .05) except ANN/MLP, ENF model fitted, and PCM.’

