# Supplementary material for: Using artificial intelligence to predict mortality in AKI patients: a systematic review/meta-analysis
Source: Clin Kidney J. 2024 May 17;17(6):sfae150. doi: 10.1093/ckj/sfae150 (PMC11187489; doi:10.1093/ckj/sfae150)
Supplement: sfae150_Supplemental_File [file sfae150_supplemental_file.docx]

**Supplemental Data Appendix**

**Table : Literature Search Strategy**

| Database | Pubmed | EMBASE |
| --- | --- | --- |
| Date | 1-12-2023 | 1-12-2023 |
| Strategy 1. | “Artificial Intelligence” [All Fields] | “Artificial Intelligence” [.exp] |
| 2. | “Machine Learning” [All Fields] | “Machine Learning” [.exp] |
| 3. | “Deep Learning” [All fields] | “Deep Learning” [.exp] |
| 4. | 1 OR 2 OR 3 | 1 OR 2 OR 3 |
| 5. | “Acute Kidney Injury” [All Fields] | “Acute Kidney failure” [.abstract] |
| 6. | “AKI” [All Fields] | “kidney injury” [.abstract] |
| 7. | “Acute Renal Failure” [All Fields] | 5 OR 6 |
| 8. | 5 OR 6 OR 7 | “Pediatrics” [.exp] |
| 9. | “Pediatrics” [All Fields] | “Adults” [.exp] |
| 10. | “Children” [All Fields] | 8 OR 9 |
| 11. | “Adults’ [All Fields] | 4 AND 7 AND 10 |
| 12. | 9 OR 10 OR 11 | Limit 11 to English |
| 13. | 4 AND 8 AND 12 |  |
| 14. | Limit 13 to English |  |
| 15. | Limit 14 from 2010-2023 |  |
| Results | 166 | 457 |

**Table: PICOS chart**

| Criteria | Inclusion Criteria | Exclusion Criteria |
| --- | --- | --- |
| Population | > 1 patients with in-hospital Acute Kidney Injury, All ages | Patients without a diagnosis of Acute Kidney Injury |
| Intervention | Artificial Intelligence, Machine Learning, Deep Learning, logistic regression, broad learning systems, extreme gradient boost, random forest models, support vector machines | No Artificial Intelligence, Machine Learning, Deep Learning, logistic regression, broad learning systems, extreme gradient boost, random forest models, support vector machines applied to patient profiles |
| Comparison | Other Artificial Intelligence, Machine Learning, Deep Learning Models | No other Artificial Intelligene, Machine Learning, Deep Learning Models compared/listed |
| Outcomes | Mortality incidence, model area under curve, accuracy, sensitivity, specificity, negative predictive value, positive predictive value | No follow-up reported, non-efficacy or mortality outcomes, prediction of AKI outcomes |
| Study Types | Cross sectional studies, prospective and retrospective studies, randomized clinical trial, control studies and observational studies, case reports | Systematic Reviews, Literature Reviews |

**Figure : PRISMA FLOW DIAGRAM**

**Identification of studies via databases and registers**

Records removed *before screening*:

Duplicate records removed (n =101 )

Records marked as ineligible by automation tools (n =0 )

Records removed for other reasons (n =0 )

Records identified from*:

Databases (n =623)

**Identification**

Records excluded**

(n =474 )

Recordsscreened

(n =522 )

**Screening**

Reports not retrieved

(n =3 )

Reports sought for retrieval

(n =48 )

Reports excluded:

Incomplete Mortality Data (n =23 )

Incorrect Study Design (n =14)

Reports assessed for eligibility

(n =45 )

**Included**

Reports of included studies

(n = 8)

**Table: Data Extraction**

| **study title** | **Author, year** | **Patient population** | **Sample size** | **Non-survivors** | **Survivors** | **Mortality** | **mean / Median age (y)** | **Cohorts** | **AI Model** | **Area under curve** | **Accuracy** | **Precision** | **Sensitivity** | **Specificity** | **PPV** | **NPV** | **F1-score** |
| --- | --- | --- | --- | --- | --- | --- | --- | --- | --- | --- | --- | --- | --- | --- | --- | --- | --- |
| Machine learning algorithm to predict mortality in patients undergoing continuous renal replacement therapy | Kang MW et al., 2020 | CRRT for acute kidney injury | 1571 | 1019 | 552 | 64.9% | 62.6 | derivation cohort | APACHE II | 0.593 (0.563–0.622) | 0.58 |  |  |  |  |  | 0.654 |
| Machine learning algorithm to predict mortality in patients undergoing continuous renal replacement therapy | Kang MW et al., 2020 | CRRT for acute kidney injury |  |  |  |  |  | derivation cohort | SOFA | 0.664 (0.636–0.691) | 0.603 |  |  |  |  |  | 0.645 |
| Machine learning algorithm to predict mortality in patients undergoing continuous renal replacement therapy | Kang MW et al., 2020 | CRRT for acute kidney injury |  |  |  |  |  | derivation cohort | MOSAIC | 0.690 (0.641–0.740) | 0.633 |  |  |  |  |  | 0.656 |
| Machine learning algorithm to predict mortality in patients undergoing continuous renal replacement therapy | Kang MW et al., 2020 | CRRT for acute kidney injury |  |  |  |  |  | derivation cohort | κ-Nearest neighbor | 0.721 (0.675–0.767) | 0.673 |  |  |  |  |  | 0.776 |
| Machine learning algorithm to predict mortality in patients undergoing continuous renal replacement therapy | Kang MW et al., 2020 | CRRT for acute kidney injury |  |  |  |  |  | derivation cohort | Support vector machine | 0.755 (0.711–0.799) | 0.686 |  |  |  |  |  | 0.782 |
| Machine learning algorithm to predict mortality in patients undergoing continuous renal replacement therapy | Kang MW et al., 2020 | CRRT for acute kidney injury |  |  |  |  |  | derivation cohort | Multivariate adaptive regression splines | 0.756 (0.713–0.799) | 0.694 |  |  |  |  |  | 0.781 |
| Machine learning algorithm to predict mortality in patients undergoing continuous renal replacement therapy | Kang MW et al., 2020 | CRRT for acute kidney injury |  |  |  |  |  | derivation cohort | Random forest | 0.768 (0.726–0.810) | 0.7 |  |  |  |  |  | 0.757 |
| Machine learning algorithm to predict mortality in patients undergoing continuous renal replacement therapy | Kang MW et al., 2020 | CRRT for acute kidney injury |  |  |  |  |  | derivation cohort | Extreme gradient boost | 0.754 (0.709–0.798) | 0.711 |  |  |  |  |  | 0.79 |
| Machine learning algorithm to predict mortality in patients undergoing continuous renal replacement therapy | Kang MW et al., 2020 | CRRT for acute kidney injury |  |  |  |  |  | derivation cohort | Artificial neural network | 0.762 (0.719–0.806) | 0.707 |  |  |  |  |  | 0.79 |
| Predicting in-hospital mortality of patients with acute kidney injury in the ICU using random forest model. | Lin K et al., 2019 | acute kidney injury in the ICU | 19044 | 2590 | 16454 | 13.6% | 63.75 | derivation cohort | Random forest | 0.866 (0.862-0.870) | 0.728 (0.715-0.741) |  |  |  |  |  | 0.459 (0.449-0.470) |
| Predicting in-hospital mortality of patients with acute kidney injury in the ICU using random forest model. | Lin K et al., 2019 | acute kidney injury in the ICU |  |  |  |  |  | derivation cohort | Support vector machine | 0.861 (0.855-0.868) | 0.729 (0.713-0.745) |  |  |  |  |  | 0.460 (0.445-0.474) |
| Predicting in-hospital mortality of patients with acute kidney injury in the ICU using random forest model. | Lin K et al., 2019 | acute kidney injury in the ICU |  |  |  |  |  | derivation cohort | Artificial neural network | 0.833 (0.818-0.848) | 0.666 (0.626-0.705) |  |  |  |  |  | 0.406 (0.381-0.437) |
| Predicting in-hospital mortality of patients with acute kidney injury in the ICU using random forest model. | Lin K et al., 2019 | acute kidney injury in the ICU |  |  |  |  |  | derivation cohort | Customized Simplified Acute Physiology Score II (SAPS II) | 0.795 (0.781-0.809) | 0.580 (0.538-0.621) |  |  |  |  |  | 0.355 (0.333-0.377) |
| Prediction of Mortality and Major Adverse Kidney Events in Critically Ill Patients With Acute Kidney Injury. | Neyra JA et al., 2022 | critically ill patients with incident AKI | 7354 | 1610 | 5744 | 21.9% | 63.02 | derivation cohort | proposed clinical models | 0.79 (0.79 - 0.80) | 0.71 (0.71 - 0.71) |  | 0.72 (0.72 - 0.73) | 0.71 (0.70 - 0.71) | 0.62 (0.59-0.65) | 0.90 (0.90 - 0.90) |  |
| Prediction of Mortality and Major Adverse Kidney Events in Critically Ill Patients With Acute Kidney Injury. | Neyra JA et al., 2022 | critically ill patients with incident AKI | 2233 | 221 | 2012 | 9.9% | 63.98 | validation cohort | proposed clinical models | 0.74 (0.73 - 0.74) | 0.65 (0.64 - 0.66) |  | 0.69 (0.67 - 0.71) | 0.64 (0.63 - 0.65) | 0.18 (0.17 - 0.18) | 0.95 (0.95 - 0.95) |  |
| Prediction of Mortality and Major Adverse Kidney Events in Critically Ill Patients With Acute Kidney Injury. | Neyra JA et al., 2022 | critically ill patients with incident AKI | 7354 | 1610 | 5744 | 21.9% | 63.02 | derivation cohort | logistic regression model | 0.77 (0.77-0.77) | 0.72 (0.72-0.72) |  | 0.67 (0.66-0.68) | 0.73 (0.73-0.74) | 0.41 (0.41-0.42) | 0.89 (0.89-0.89 |  |
| Prediction of Mortality and Major Adverse Kidney Events in Critically Ill Patients With Acute Kidney Injury. | Neyra JA et al., 2022 | critically ill patients with incident AKI | 7354 | 1610 | 5744 | 21.9% | 63.02 | derivation cohort | Random forest | 0.79 (0.79-0.80) | 0.71 (0.71-0.71) |  | 0.72 (0.72-0.73) | 0.71 (0.70-0.71 | 0.41 (0.40-0.42) | 0.90 (0.90-0.90) |  |
| Prediction of Mortality and Major Adverse Kidney Events in Critically Ill Patients With Acute Kidney Injury. | Neyra JA et al., 2022 | critically ill patients with incident AKI | 7354 | 1610 | 5744 | 21.9% | 63.02 | derivation cohort | Support vector machine | 0.77 (0.77-0.77 | 0.72 (0.72-0.72) |  | 0.67 (0.66-0.67) | 0.74 (0.73-0.74) | 0.41 (0.41-0.42) | 0.89 (0.89-0.89) |  |
| Prediction of Mortality and Major Adverse Kidney Events in Critically Ill Patients With Acute Kidney Injury. | Neyra JA et al., 2022 | critically ill patients with incident AKI | 7354 | 1610 | 5744 | 21.9% | 63.02 | derivation cohort | Extreme gradient boost | 0.78 (0.78-0.78) | 0.71 (0.71-0.71) |  | 0.68 (0.68-0.69) | 0.72 (0.71-0.72) | 0.41 (0.40-0.41) | 0.89 (0.89-0.89) |  |
| Prediction of Mortality and Major Adverse Kidney Events in Critically Ill Patients With Acute Kidney Injury. | Neyra JA et al., 2022 | critically ill patients with incident AKI | 2233 | 221 | 2012 | 9.9% | 63.98 | validation cohort | logistic regression model | 0.77 (0.77-0.78) | 0.64 (0.62-0.66) |  | 0.77 (0.74-0.79) | 0.63 (0.60-0.65) | 0.19 (0.18-0.19) | 0.96 (0.96-0.96) |  |
| Prediction of Mortality and Major Adverse Kidney Events in Critically Ill Patients With Acute Kidney Injury. | Neyra JA et al., 2022 | critically ill patients with incident AKI | 2233 | 221 | 2012 | 9.9% | 63.89 | validation cohort | Random forest | 0.74 (0.73-0.74) | 0.65 (0.64-0.66) |  | 0.69 (0.67-0.71) | 0.64 (0.63-0.65) | 0.18 (0.17-0.18) | 0.95 (0.95-0.95) |  |
| Prediction of Mortality and Major Adverse Kidney Events in Critically Ill Patients with Acute Kidney Injury. | Neyra JA et al., 2022 | critically ill patients with incident AKI | 2233 | 221 | 2012 | 9.9% | 63.98 | validation cohort | Support vector machine | 0.78 (0.77-0.79) | 0.64 (0.62-0.66) |  | 0.79 (0.76-0.81) | 0.62 (0.59-0.65) | 0.19 (0.18-0.20) | 0.96 (0.96-0.97) |  |
| Prediction of Mortality and Major Adverse Kidney Events in Critically Ill Patients With Acute Kidney Injury. | Neyra JA et al., 2022 | critically ill patients with incident AKI | 2233 | 221 | 2012 | 9.9% | 63.98 | validation cohort | Extreme gradient boost | 0.70 (0.68-0.72) | 0.62 (0.60-0.65) |  | 0.67 (0.64-0.69) | 0.62 (0.59-0.65) | 0.16 (0.15-0.17) | 0.94 (0.94-0.95) |  |
| Approaches to Predicting Outcomes in Patients with Acute Kidney Injury | Saly D et al., 2017 | Patients with Acute Kidney Injury | 1098 | 108 | 990 | 9.8% | 62.1 | derivation cohort | logistic regression model | 0.90 (0.88-0.93) |  |  |  |  |  |  |  |
| Approaches to Predicting Outcomes in Patients with Acute Kidney Injury | Saly D et al., 2017 | Patients with Acute Kidney Injury | 2233 | 221 | 2012 | 9.9% | 63.98 | derivation cohort | Random forest | 0.85 (0.81-0.90) |  |  |  |  |  |  |  |
| Approaches to Predicting Outcomes in Patients with Acute Kidney Injury | Saly D et al., 2017 | Patients with Acute Kidney Injury | 1143 | 112 | 1031 | 9.8% | 62.8 | validation cohort | logistic regression model | 0.80 (0.75-0.84) |  |  |  |  |  |  |  |
| Approaches to Predicting Outcomes in Patients with Acute Kidney Injury | Saly D et al., 2017 | Patients with Acute Kidney Injury | 2233 | 221 | 2012 | 9.9% | 63.98 | validation cohort | Random forest | 0.80 (0.76-0.85) |  |  |  |  |  |  |  |
| Development and validation of prediction model using nursing notes on sentiment scores for prognosis of patients with severe acute kidney injury receiving continuous renal replacement therapy based on computational intelligence algorithms | Zha D et al., 2022 | AKI patients undergoing CRRT | 189 | 97 | 92 | 51.3% | 63.4 | Derivation | Random forest [with sentiment scores] | 0.86 (0.81–0.91) | 0.79 (0.73–0.85) | 0.78 (0.68–0.88) | 0.72 (0.63–0.80) | 0.87 (0.80–0.94) | 0.87 (0.80–0.94) | 0.72 (0.64–0.81) |  |
| Development and validation of prediction model using nursing notes on sentiment scores for prognosis of patients with severe acute kidney injury receiving continuous renal replacement therapy based on computational intelligence algorithms | Zha D et al., 2022 | AKI patients undergoing CRRT | 7354 | 1610 | 5744 | 21.9% | 63.02 | Derivation | broad learning system (BLS) models (with sentiment scores) | 0.87 (0.82–0.92) | 0.74 (0.67–0.80) | 0.82 (0.73–0.91) | 0.95 (0.91–0.99) | 0.48 (0.38–0.59) | 0.68 (0.60–0.77) | 0.89 (0.81–0.98) |  |
| Development and validation of prediction model using nursing notes on sentiment scores for prognosis of patients with severe acute kidney injury receiving continuous renal replacement therapy based on computational intelligence algorithms | Zha D et al., 2022 | AKI patients undergoing CRRT | 7354 | 1610 | 5744 | 21.9% | 63.02 | Derivation | Random forest [without sentiment scores] | 0.85 (0.80–0.90) | 0.78 (0.72–0.84) |  | 0.91 (0.86–0.97) | 0.63 (0.53–0.73) | 0.74 (0.67–0.82) | 0.86 (0.77–0.94) |  |
| Development and validation of prediction model using nursing notes on sentiment scores for prognosis of patients with severe acute kidney injury receiving continuous renal replacement therapy based on computational intelligence algorithms | Zha D et al., 2022 | AKI patients undergoing CRRT | 7354 | 1610 | 5744 | 21.9% | 63.02 | Derivation | broad learning system (BLS) models (without sentiment scores) | 0.87 (0.82–0.92) | 0.78 (0.72–0.84) |  | 0.84 (0.77–0.91) | 0.71 (0.62–0.81) | 0.77 (0.70–0.85) | 0.79 (0.71–0.88) |  |
| Development and validation of prediction model using nursing notes on sentiment scores for prognosis of patients with severe acute kidney injury receiving continuous renal replacement therapy based on computational intelligence algorithms | Zha D et al., 2022 | AKI patients undergoing CRRT | 81 | 42 | 39 | 51.9% | 63.4 | validation cohort | Random forest [with sentiment scores] | 0.78 (0.68–0.88) | 0.70 (0.60–0.80) |  | 0.65 (0.49–0.80) | 0.75 (0.62–0.88) | 0.69 (0.53–0.84) | 0.72 (0.59–0.85) |  |
| Development and validation of prediction model using nursing notes on sentiment scores for prognosis of patients with severe acute kidney injury receiving continuous renal replacement therapy based on computational intelligence algorithms | Zha D et al., 2022 | AKI patients undergoing CRRT | 2233 | 221 | 2012 | 9.9% | 63.98 | validation cohort | broad learning system (BLS) models (with sentiment scores) | 0.82 (0.73–0.91) | 0.72 (0.62-0.81) |  | 0.41 (0.25–0.56) | 0.98 (0.93–1.00) | 0.94 (0.82–1.00) | 0.66 (0.55–0.78) |  |
| Development and validation of prediction model using nursing notes on sentiment scores for prognosis of patients with severe acute kidney injury receiving continuous renal replacement therapy based on computational intelligence algorithms | Zha D et al., 2022 | AKI patients undergoing CRRT | 2233 | 221 | 2012 | 9.9% | 63.98 | validation cohort | Random forest [without sentiment scores] | 0.72 (0.60–0.83) | 0.62 (0.51–0.72) |  | 0.81 (0.68–0.94) | 0.45 (0.31–0.60) | 0.56 (0.42–0.69) | 0.74 (0.58–0.91) |  |
| Development and validation of prediction model using nursing notes on sentiment scores for prognosis of patients with severe acute kidney injury receiving continuous renal replacement therapy based on computational intelligence algorithms | Zha D et al., 2022 | AKI patients undergoing CRRT | 2233 | 221 | 2012 | 9.9% | 63.98 | validation cohort | broad learning system (BLS) models (without sentiment scores) | 0.75 (0.64–0.85) | 0.67 (0.56–0.77) |  | 0.59 (0.44–0.75) | 0.73 (0.60–0.86) | 0.65 (0.49–0.81) | 0.68 (0.55–0.81) |  |
| Development of a prediction score for in‐hospital mortality in COVID‐19 patients with acute kidney injury: a machine learning approach | Ponce D et al., 2021 | in-hospital mortality in COVID-19 patients with AKI | 697 | 436 | 261 | 62.6% | 63 | Derivation | Random forest | 0.894 (0.82-0.93) |  |  |  |  |  |  |  |
| Development of a prediction score for in‐hospital mortality in COVID‐19 patients with acute kidney injury: a machine learning approach | Ponce D et al., 2021 | in-hospital mortality in COVID-19 patients with AKI |  |  |  |  |  | Derivation | Extreme gradient boost | 0.886 (0.85-0.95) |  |  |  |  |  |  |  |
| Development of a prediction score for in‐hospital mortality in COVID‐19 patients with acute kidney injury: a machine learning approach | Ponce D et al., 2021 | in-hospital mortality in COVID-19 patients with AKI | 1143  173 | 112  108 | 1031  65 | 9.8%  62.4% | 62.8  63 | Derivation | Elastic Net final model fitted | 0.877 (0.83-0.93) |  |  |  |  |  |  |  |
| Development of a prediction score for in‐hospital mortality in COVID‐19 patients with acute kidney injury: a machine learning approach | Ponce D et al., 2021 | in-hospital mortality in COVID-19 patients with AKI |  |  |  |  |  | validation cohort | Random Forest | 0.831 (0.76-0.89) |  |  |  |  |  |  |  |
| Development of a prediction score for in‐hospital mortality in COVID‐19 patients with acute kidney injury: a machine learning approach | Ponce D et al., 2021 | in-hospital mortality in COVID-19 patients with AKI | 189  2666 | 97  790 | 92  1876 | 51.3%  29.6% | 63.4  72.7 | validation cohort | Extreme gradient boost | 0.823 (0.75-0.88) |  |  |  |  |  |  |  |
| Development of a prediction score for in‐hospital mortality in COVID‐19 patients with acute kidney injury: a machine learning approach | Ponce D et al., 2021 | in-hospital mortality in COVID-19 patients with AKI |  |  |  |  |  | validation cohort | Elastic Net final model fitted | 0.821 (0.75-0.88) |  |  |  |  |  |  |  |
| Development and deployment of interpretable machine-learning model for predicting in-hospital mortality in elderly patients with acute kidney disease. | Li M et al., 2022 | in-hospital mortality in elderly patients with acute kidney disease |  |  |  |  |  | Derivation | logistic regression model | 0.812 (0.793–0.832) |  |  | 0.673 (0.639–0.706) | 0.809 (0.790–0.826) | 0.597 (0.564–0.629) | 0.855 (0.837–0.871) |  |
| Development and deployment of interpretable machine-learning model for predicting in-hospital mortality in elderly patients with acute kidney disease. | Li M et al., 2022 | in-hospital mortality in elderly patients with acute kidney disease |  |  |  |  |  | Derivation | Random forest | 0.855 (0.838–0.873) |  |  | 0.697 (0.663–0.728) | 0.845 (0.828–0.861) | 0.653 (0.621–0.686) | 0.868 (0.852–0.884) |  |
| Development and deployment of interpretable machine-learning model for predicting in-hospital mortality in elderly patients with acute kidney disease. | Li M et al., 2022 | in-hospital mortality in elderly patients with acute kidney disease | 81 | 42 | 39 | 51.9% | 63.4 | Derivation | Extreme gradient boost | 0.899 (0.884–0.914) |  |  | 0.799 (0.769–0.826) | 0.830 (0.812–0.847) | 0.664 (0.633–0.694) | 0.907 (0.893–0.921) |  |
| Development and deployment of interpretable machine-learning model for predicting in-hospital mortality in elderly patients with acute kidney disease. | Li M et al., 2022 | in-hospital mortality in elderly patients with acute kidney disease |  |  |  |  |  | Derivation | multilayer perceptron (MLP) | 0.824 (0.805–0.843) |  |  | 0.675 (0.641–0.707) | 0.821 (0.803–0.838) | 0.613 (0.580–0.646) | 0.857 (0.840–0.873) |  |
| Development and deployment of interpretable machine-learning model for predicting in-hospital mortality in elderly patients with acute kidney disease. | Li M et al., 2022 | in-hospital mortality in elderly patients with acute kidney disease |  |  |  |  |  | Derivation | Support vector machine | 0.823 (0.804–0.842) |  |  | 0.722 (0.689–0.753) | 0.787 (0.768–0.806) | 0.588 (0.557–0.619) | 0.870 (0.853–0.886) |  |
| Development and deployment of interpretable machine-learning model for predicting in-hospital mortality in elderly patients with acute kidney disease. | Li M et al., 2022 | in-hospital mortality in elderly patients with acute kidney disease |  |  |  |  |  | Derivation | Simplified SVM | 0.810 (0.790–0.830) |  |  | 0.713 (0.680–0.744) | 0.768 (0.748–0.787) | 0.564 (0.532–0.595) | 0.864 (0.846–0.880) |  |
| Development and deployment of interpretable machine-learning model for predicting in-hospital mortality in elderly patients with acute kidney disease. | Li M et al., 2022 | in-hospital mortality in elderly patients with acute kidney disease | 535 | 172 | 363 | 32.1% | 77.7 | validation cohort | logistic regression model | 0.739 (0.691–0.786) |  |  | 0.634 (0.557–0.706) | 0.733 (0.684–0.778) | 0.529 (0.459–0.599) | 0.809 (0.762–0.850) |  |
| Development and deployment of interpretable machine-learning model for predicting in-hospital mortality in elderly patients with acute kidney disease. | Li M et al., 2022 | in-hospital mortality in elderly patients with acute kidney disease |  |  |  |  |  | validation cohort | Random forest | 0.689 (0.639–0.739) |  |  | 0.663 (0.587–0.733) | 0.651(0.599–0.699) | 0.473 (0.409–0.538) | 0.803 (0.753–0.847) |  |
| Development and deployment of interpretable machine-learning model for predicting in-hospital mortality in elderly patients with acute kidney disease. | Li M et al., 2022 | in-hospital mortality in elderly patients with acute kidney disease |  |  |  |  |  | validation cohort | Extreme gradient boost | 0.716 (0.668–0.765) |  |  | 0.622 (0.545–0.695) | 0.738 (0.690–0.783) | 0.530 (0.458–0.600) | 0.805 (0.758–0.846) |  |
| Development and deployment of interpretable machine-learning model for predicting in-hospital mortality in elderly patients with acute kidney disease. | Li M et al., 2022 | in-hospital mortality in elderly patients with acute kidney disease | 173 | 108 | 65 | 62.4% | 63 | validation cohort | multilayer perceptron (MLP) | 0.731 (0.683–0.779) |  |  | 0.610 (0.533–0.684) | 0.755(0.707–0.798) | 0.541 (0.468–0.613) | 0.804 (0.757–0.844) |  |
| Development and deployment of interpretable machine-learning model for predicting in-hospital mortality in elderly patients with acute kidney disease. | Li M et al., 2022 | in-hospital mortality in elderly patients with acute kidney disease |  |  |  |  |  | validation cohort | Support vector machine | 0.759 (0.713–0.805) |  |  | 0.634 (0.557–0.706) | 0.796 (0.751–0.836) | 0.595 (0.521–0.667) | 0.821 (0.777–0.860) |  |
| Development and deployment of interpretable machine-learning model for predicting in-hospital mortality in elderly patients with acute kidney disease. | Li M et al., 2022 | in-hospital mortality in elderly patients with acute kidney disease |  |  |  |  |  | validation cohort | Simplified SVM | 0.776 (0.731–0.821) |  |  | 0.738 (0.666–0.802) | 0.686 (0.635–0.733) | 0.527 (0.462–0.591) | 0.846 (0.801–0.886) |  |
| A novel predictive model for poor in-hospital outcomes in patients with acute kidney injury after cardiac surgery | Chen Z et al., 2021 | cardiac surgery-associated acute kidney injury | 196  52 | 32  11 | 164  41 | 16.3%  21.2% |  | Derivation | logistic regression model | 0.947 (0.895-0.998) |  |  | 0.969 | 0.872 | 0.596 | 0.993 |  |
| A novel predictive model for poor in-hospital outcomes in patients with acute kidney injury after cardiac surgery | Chen Z et al., 2021 | cardiac surgery-associated acute kidney injury |  |  |  |  |  | validation | logistic regression model | 0.971 (0.932-1.000) |  |  | 0.727 | 0.951 | 0.8 | 0.929 |  |

**Table: Newcastle-Ottawa Risk of Bias – Quality Assessment**

| Cohort Studies |  | Selection | | | | Comparability | Exposure | | |  |
| --- | --- | --- | --- | --- | --- | --- | --- | --- | --- | --- |
| First Author | Year | Representativeness of exposed cohort | Selection of the non-exposed cohort | Ascertainment of exposure | Demonstration that outcome of interest was not present at start of study | Comparability of cohorts based on the design or analysis | Assessment of outcome | Was follow-up long enough for outcomes to occur | Adequacy of follow up cohorts | Quality Score |
| Kang MW et al., 2020^5^ | 2022 | 1 | 1 | 1 | 1 | 2 | 1 | 1 | 1 | 9/9 |
| Lin K et al., 2019^7^ | 2020 | 1 | 1 | 1 | 1 | 1 | 1 | 1 | 1 | 8/9 |
| Neyra JA et al., 2022^11^ | 2020 | 1 | 1 | 1 | 1 | 2 | 1 | 1 | 1 | 9/9 |
| Saly D et al., 2017^6^ | 2019 | 1 | 1 | 1 | 1 | 2 | 1 | 1 | 1 | 9/9 |
| Zha D et al., 2022^4^ | 2019 | 1 | 1 | 1 | 1 | 2 | 1 | 1 | 1 | 9/9 |
| Ponce D et al., 2021^10^ | 2019 | 1 | 1 | 1 | 1 | 2 | 1 | 1 | 1 | 9/9 |
| Li M et al., 2022^9^ | 2018 | 1 | 1 | 1 | 1 | 1 | 1 | 1 | 1 | 8/9 |
| Chen Z et al., 2021^8^ | 2016 | 1 | 1 | 1 | 1 | 1 | 1 | 1 | 1 | 8/9 |

Figure: Forest plot of the meta-analysis of logistic regression model area under the curve across different studies. The lower diamond in the graph represents the pooled estimate

Figure: Forest plot of the meta-analysis of extreme gradient boost model area under the curve across different studies. The lower diamond in the graph represents the pooled estimate

**Figure:** Forest plot of the meta-analysis of random forest model area under the curve across different studies. The lower diamond in the graph represents the pooled estimate

**Figure:**  Forest plot of the meta-analysis of support vector machine model area under the curve across different studies. The lower diamond in the graph represents the pooled estimate

**Figure:** Forest plot of the meta-analysis of artificial neural network / multi-layer perceptron area under the curve across different studies. The lower diamond in the graph represents the pooled estimate

**Figure:** Forest plot of the meta-analysis of broad learning system (BLS) models area under the curve across different studies. The lower diamond in the graph represents the pooled estimate

**Figure:** Forest plot of the meta-analysis of elastic net final model fitted models area under the curve across different studies. The lower diamond in the graph represents the pooled estimate

**Figure:** Forest plot of the meta-analysis of proposed clinical model area under the curve across different studies. The lower diamond in the graph represents the pooled estimate

**Table: AMSTAR Systematic Review Criteria Checklist, and PRISMA FLOW Checklist:**

| Were the PICO components included? | yes |
| --- | --- |
| Was a prior design provided | yes |
| Did the author explain their selection of study designs for inclusion in the review | yes |
| Did the review authors use a comprehensive literature search strategy? | yes |
| Did the review authors perform study selection in duplicate? | yes |
| Did the review authors perform data extraction in duplicate? | yes |
| Did the review authors provide a list of excluded studies and justify the exclusions? | yes |
| Did the review authors describe the included studies in adequate detail? | yes |
| Satisfactory technique to assess RoB was used? | yes |
| Did the review authors report on the sources of funding for the studies included in the review? | no |
| Appropriate methods for statistical combination of results was used for included studies? | yes |
| Review authors assessed the potential impact of RoB in individual studies on the results of the meta-analysis | yes |
| Did the review authors account for RoB in individual studies when interpreting/discussing the results of the review | yes |
| Did the review authors provide a satisfactory explanation for any heterogeneity observed in the results of the review? | yes |
| Was publication bias investigated and its impact on the results of the review? | yes |
| Total score: | 13/14 |
